# Azithromycin, a potent autophagy inhibitor for cancer therapy, perturbs cytoskeletal protein dynamics

**DOI:** 10.1038/s41416-023-02210-4

**Published:** 2023-03-04

**Authors:** Naoharu Takano, Masaki Hiramoto, Yumiko Yamada, Hiroko Kokuba, Mayumi Tokuhisa, Hirotsugu Hino, Keisuke Miyazawa

**Affiliations:** 1 Department of Biochemistry, Tokyo Medical University, Tokyo, Japan; 2 Laboratory of Electron Microscopy, Tokyo Medical University, Tokyo, Japan

**Keywords:** Drug development, Macroautophagy, Target identification, Lysosomes

## Abstract

**Background:**

Autophagy plays an important role in tumour cell growth and survival and also promotes resistance to chemotherapy. Hence, autophagy has been targeted for cancer therapy. We previously reported that macrolide antibiotics including azithromycin (AZM) inhibit autophagy in various types of cancer cells in vitro. However, the underlying molecular mechanism for autophagy inhibition remains unclear. Here, we aimed to identify the molecular target of AZM for inhibiting autophagy.

**Methods:**

We identified the AZM-binding proteins using AZM-conjugated magnetic nanobeads for high-throughput affinity purification. Autophagy inhibitory mechanism of AZM was analysed by confocal microscopic and transmission electron microscopic observation. The anti-tumour effect with autophagy inhibition by oral AZM administration was assessed in the xenografted mice model.

**Results:**

We elucidated that keratin-18 (KRT18) and α/β-tubulin specifically bind to AZM. Treatment of the cells with AZM disrupts intracellular KRT18 dynamics, and *KRT18* knockdown resulted in autophagy inhibition. Additionally, AZM treatment suppresses intracellular lysosomal trafficking along the microtubules for blocking autophagic flux. Oral AZM administration suppressed tumour growth while inhibiting autophagy in tumour tissue.

**Conclusions:**

As drug-repurposing, our results indicate that AZM is a potent autophagy inhibitor for cancer treatment, which acts by directly interacting with cytoskeletal proteins and perturbing their dynamics.

## Background

Autophagy, a lysosome-dependent system, degrades cellular components; it is involved in protein degradation to remove or recycle amino acids, thereby supplying energy under starvation conditions, and helps degrade damaged organelles for cellular homoeostasis [1]. Autophagy is involved in various aspects of cancer cell biology, e.g., carcinogenesis, tumour development, cancer stem cell maintenance, and resistance to chemotherapeutics [2–5]. Autophagy-deficient mice that are systematically mosaic for *Atg5* deficiency, or are liver-specific *Atg7* knockouts, develop liver adenomas, indicating that autophagy suppresses carcinogenesis [6]. In contrast, autophagy promotes tumorigenesis by supporting cancer cell proliferation and tumour growth. Hence, inhibiting autophagy suppresses tumour development [2]. Suppressing autophagy in non-malignant host cells, including those in the tumour microenvironment, suppresses tumour growth [7–10]. Small molecule inhibitors of autophagy can suppress tumour growth both in vitro and in vivo [11]. Thus, autophagy inhibitors may be suitable for cancer therapy; however, the only clinically available autophagy inhibitors are chloroquine (CQ) and hydroxychloroquine (HCQ). Several ongoing clinical trials involve HCQ administration in combination with other anti-cancer drugs. Although some prior trials showed a promising effect, not all patients were deemed fit to receive sufficient HCQ (1200 mg/day), owing to adverse events [12, 13]. Therefore, the development of an effective and feasible autophagy inhibitor with lower toxicity is desirable in the clinical setting.

Macrolide antibiotics, including azithromycin (AZM) and clarithromycin (CAM), inhibit autophagy [14–16]. Combined treatment with a proteasome inhibitor and macrolide resulted in pronounced cytotoxicity in myeloma and breast cancer cells, along with endoplasmic reticulum (ER) stress loading, owing to simultaneous inhibition of two major protein degradation systems: autophagy-lysosome system and ubiquitin-proteasomal system [17, 18]. Tyrosine kinase inhibitors (TKIs), including imatinib, dasatinib, gefitinib, erlotinib, and sorafenib, induce cytoprotective autophagy [19, 20]. Hence, co-treatment with these TKIs and macrolide for blocking autophagy has led to enhanced cytotoxic effects [21–23]. Since macrolide antibiotics exhibit almost no cytotoxicity alone, they appear to function as an adjuvant for TKIs in cancer treatment. Furthermore, the cytotoxic activities of DNA‐damaging drugs (e.g., doxorubicin, etoposide, and carboplatin) were enhanced in the presence of AZM in non-small cell lung cancer cell lines [24]. DNA-damaging drugs caused lysosomal damage and upregulated lysosomal biogenesis. Simultaneously, AZM treatment blocked autophagy to eliminate impaired lysosomes and accumulate lysosomes/autolysosomes. Thus, the effects were integrated into the marked increase in the damaged lysosomes/autolysosomes, leading to prominent lysosomal membrane permeabilization (LMP) for apoptosis induction.

Therefore, macrolide antibiotics can potentially be used as autophagy inhibitors in cancer therapy. However, the underlying molecular mechanism of AZM for autophagy inhibition is unclear. In the present study, we aimed to identify its molecular target during autophagy inhibition, using AZM-conjugated magnetic nanobeads for high-throughput affinity purification [25].

## Methods

We treated human lung cancer (A549), human multiple myeloma (IM-9), and adrenal cortex-derived human adenocarcinoma (SW13) cell lines with/without AZM, and performed autophagic flux assay through western blotting using anti-LC3B antibody. We also used A549 cells expressing GFP-LC3/RFP-LC3ΔG for autophagic flux assay with a real-time live-cell imaging system. AZM binding proteins were identified using high-throughput affinity purification with AZM-conjugated magnetic nanobeads followed by LC-MS/MS analysis. We also established cell lines stably expressing the fluorescent proteins: A549/AcGFP-KRT18, A549/LAMP1-EGFP, A549/mCherry-LC3, A549/AcGFP-αTubulin, A549/mCherry-EGFP-LC3, SW13/LAMP1-EGFP, and SW13/mCherry-EGFP-LC3. To analyse the autophagosome/autolysosome formation and lysosomal maturation process, we examined data from immunofluorescence staining, live-cell imaging, and transmission electron microscopy. In vivo tumour growth inhibitory effects of AZM were determined in a xenografted mice model. OligoDNA sequences used for shRNA construction are listed in Table S1, and their details are provided in Supplementary Materials and Methods.

## Results

### AZM inhibits both autophagy and tumour growth

**Fig. 1:**
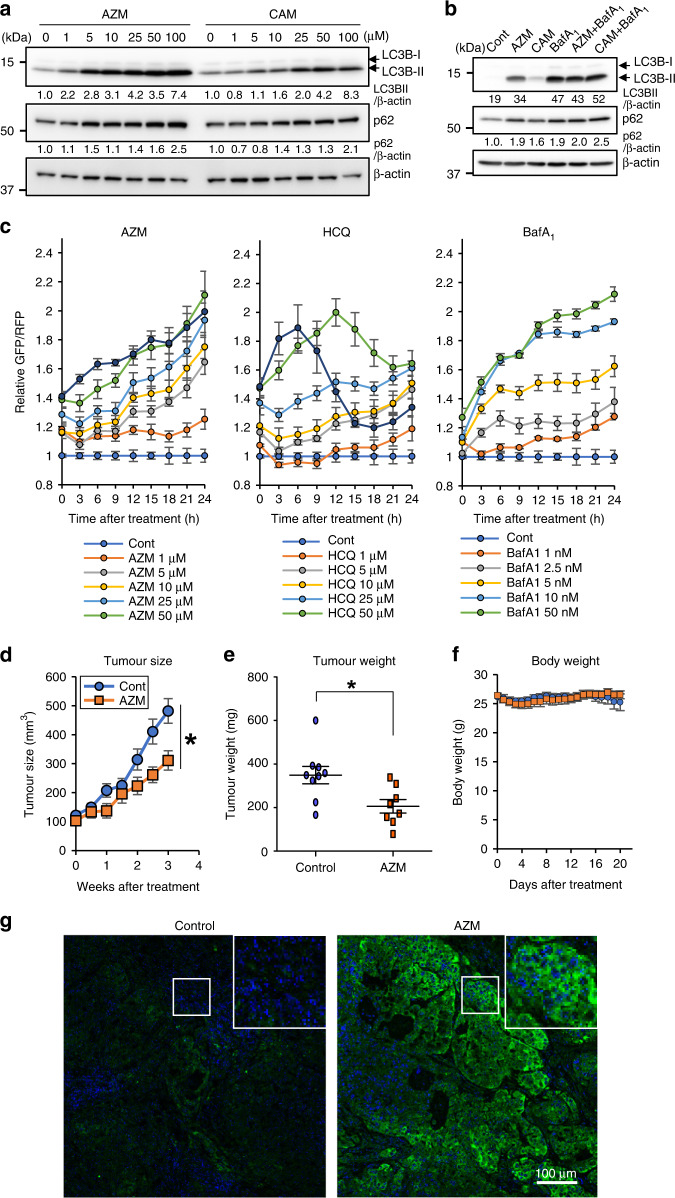
AZM suppresses autophagy in vitro and in vivo. **a** A549 cells were treated with AZM/CAM at various concentrations for 24 h. LC3B and p62 expression levels were determined via western blotting. The band intensity of LC3B-II and p62 were standardised with β-actin and a relative value was shown below each panel. **b** For autophagic flux assay, A549 cells were treated with either 50 μM AZM or CAM ± 10 nM BafA_1_. **c** A549 cells expressing GFP-LC3/RFP-LC3ΔG were treated with AZM, HCQ, or BafA_1_ for 24 h, and fluorescence intensity was monitored with an IncuCyte ZOOM real-time live-cell imaging system. Calculated relative GFP/RFP ratio to control treated cells were shown. *n* = 4, bar = mean ± SE (**d**–**f**) A549 cells were implanted into the flank of immunodeficient mice and treated daily with AZM (100 μg/g/day) or vehicle, via oral gavage. Tumour size was measured twice a week, while body weight was measured daily. After 3 weeks, tumour tissues were harvested, weighed, and used following immunohistochemistry. *n* = 9, control and *n* = 8, AZM. Bar = mean ± SE, *p < 0.05. **g** p62 expression was determined using immunofluorescence staining (green). Nuclei were stained with DAPI (blue). Scale = 100 μm.

Macrolide antibiotics have previously been shown to inhibit autophagy [15, 16, 21]. To determine the potential of macrolide antibiotics for treating cancer, we assessed the autophagy inhibitory effect of AZM and CAM in A549 cells. AZM (>5 μM) increased levels of both the autophagosomal marker LC3B-II and autophagic substrate p62 in a dose-dependent manner, indicating autophagy inhibition (Fig. 1a). AZM treatment was accompanied by a greater increase in LC3B-II and p62 levels, compared with CAM treatment (Fig. 1a). To study autophagic flux, we co-administered bafilomycin A1 (BafA_1_) with either AZM or CAM. There was no further increase in LC3B-II or p62 levels compared with BafA_1_ administration alone. Moreover, co-administration of BafA_1_ with AZM or CAM induces higher LC3B-II levels than AZM or CAM administration alone. This indicates that although AZM and CAM block autophagic flux, some residual autophagic flux remained when cells were treated with AZM or CAM alone (Fig. 1b) [26]. Since AZM was a more potent autophagy inhibitor than CAM, we used AZM in our experiments.

Next, we compared autophagy inhibitory activity and cell toxicity of AZM with those of other well-known autophagy inhibitors, HCQ and BafA_1_ [27, 28], using an autophagic flux probe (GFP-LC3/RFP-LC3ΔG) [29]; GFP-LC3 and RFP-LC3ΔG are expressed at equal levels in transfected cells, but GFP-LC3 is involved in isolation membrane growth to form autophagosomes, and fusion with lysosome quenches the GFP signal due to its acidic environment, and GFP-LC3 is subsequently degraded via autophagy. In contrast, RFP-LC3ΔG remains in the cytosol because it lacks the glycine residue involved in autophagosome formation and serves as an internal control. Therefore, GFP/RFP ratios represent autophagic flux [29]. We analysed the cell confluency, GFP, and RFP signals separately (Fig. S1).

AZM treatment increased the GFP/RFP ratio (>5 μM) meaning inhibition of autophagy, as same as immunoblotting data, and the GFP/RFP ratio increased in a dose-dependent manner; HCQ and BafA_1_ treatment yielded similar results (Fig. 1c). Cell growth inhibition upon AZM treatment was moderate, even at higher concentrations, compared with HCQ treatment (Fig. S1a). Although HCQ increased the GFP/RFP ratio in a dose-dependent manner, this ratio decreased after 6–12 h at higher concentrations. This decrease probably occurred due to strong cell toxicity because RFP signals increased as cells began to die at higher HCQ concentrations (Fig. 1c, Fig. S1). After 24 h of exposure, AZM treatment exhibited better autophagy inhibition than HCQ autophagy inhibition (GFP/RFP ratio = 5–25 μM). BafA_1_ (≤50 nM) exhibited similar inhibitory effects as AZM after 24 h (Fig. 1c, Fig. S1). Based on this assay system, AZM inhibits autophagy to a similar extent as HCQ, but with lower cytotoxicity at equivalent concentrations in A549 cells cultured in a complete medium.

Genetic disruption of autophagy has been reported to suppress tumour growth [2]. The role of autophagy in tumour progression is to support metabolic homoeostasis of cancer cells in a harsh microenvironment [30]. We previously reported that AZM induces cell death in head and neck squamous cancer cell lines under the amino acid-depleted condition but not in the complete culture medium [31]. Therefore, we assessed whether AZM can inhibit autophagy and tumour growth in vivo, using a tumour xenograft model. Daily oral administration of AZM suppressed xenograft tumour growth and reduced harvested tumour weight without any weight loss in recipient mice (Fig. 1d–f). To determine the in vivo autophagy inhibitory effect, we performed immunofluorescence staining for p62 on harvested tumour tissues. AZM treatment increased p62 expression, suggesting that autophagy is suppressed by AZM in vivo (Fig. 1g). Our results indicate that AZM inhibits autophagy in vitro and in vivo and suppresses xenograft tumour growth when administered orally.

### AZM interacts with KRT18

**Fig. 2:**
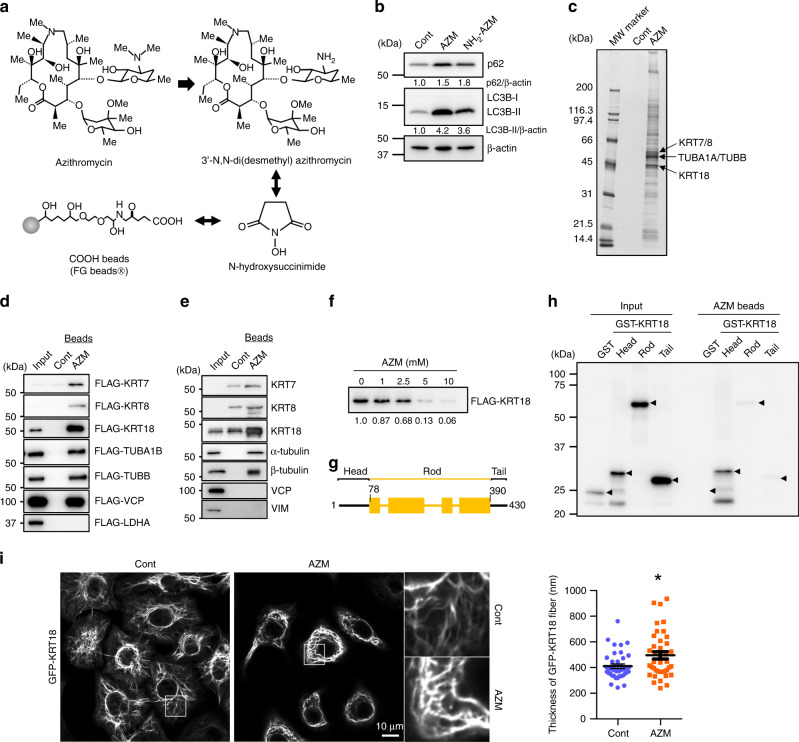
AZM interacts with KRT18 head domain and inhibits intracellular dynamics. **a** AZM-conjugated beads were prepared from 3’-N,N-Di(desmethyl)azithromycin (NH_2_-AZM) and COOH beads by crosslinking with N-hydroxysuccinimide. **b** Western blotting confirms autophagy inhibitory effect of NH_2_-AZM. A549 cells treated with 50 μM AZM or NH_2_-AZM for 24 h before being lysed. The band intensity of LC3B-II and p62 were standardised with β-actin and a relative value was shown below each panel. **c** Silver staining shows proteins bound to AZM-conjugated and control beads. Molecular marker was loaded on the left lane. Arrows indicate major bands identified as KRT7/8/18 and α/β-tubulin. **d** Interaction between AZM-conjugated beads and FLAG-tagged recombinant proteins expressed in 293 T cells, detected via western blotting. **e** Binding between endogenous proteins expressed in A549 cells and AZM-conjugated beads, as determined via western blotting with protein-specific antibodies. **f** Specificity of the interaction between FLAG-KRT18 and AZM-conjugated beads, as determined in excess AZM. Relative band intensities were shown below the panel. **g** Schematic diagram of KRT18 structure. Numbers indicate amino acid residue number. **h** Binding assay between recombinant KRT18 domains expressed in *Escherichia coli* and AZM-conjugated beads. Arrowheads indicate the size of each protein at the predicted size. **i** Confocal microscopic observation of A549 cells expressing GFP-KRT18 treated with DMSO or 50 μM AZM for 24 h. Scale = 10 μm. Enlarged view of boxed area on the right. Ten locations were randomly selected from 5 cells, and the thickness of the fibres was measured and summarised in right graph. Bar = mean ± S.E., *p < 0.05 v.s. control. Time lapse videos of these cells are in Supplementary Videos 1 and 2.

We attempted to identify the molecular target of AZM for autophagy inhibition. We generated AZM-conjugated magnetic nanobeads by crosslinking 3’-N,N-Di(desmethyl) azithromycin (NH_2_-AZM) and COOH beads (FG beads) (Fig. 2a). NH_2_-AZM is an AZM-derivative that inhibits autophagy (Fig. 2b). AZM-conjugated beads were incubated with A549 cell lysate, and proteins that bound to the beads were detected via silver staining and identified via LC-MS/MS analysis (Fig. 2c, Fig. S2, Table. S2). Notably, we identified intermediate filament protein keratin-7/8/18 (KRT7/8/18) and α/β-tubulin (TUBA1B/ TUBB) as major AZM-binding protein (Fig. 2c arrows). We found that both FLAG-tagged recombinant KRT7/8/18 and TUBA1B/TUBB and endogenous forms can bind to AZM-conjugated beads (Fig. 2d, e). Although valosin-containing protein (VCP) was previously reported as an AZM-binding protein [32], we found that only recombinant VCP can bind to AZM-conjugated beads. We used FLAG-LDH and another intermediate filament protein, vimentin (VIM), as negative controls (Fig. 2d, e). We focused on KRT18 because it was the protein most enriched by AZM-conjugated beads (Fig. 2d, e). AZM addition to our binding assay solution inhibited the interaction between FLAG-KRT18 and AZM-conjugated beads in a dose-dependent manner (Fig. 2f), indicating the specific interaction between AZM-conjugated beads and FLAG-KRT18.

KRT18 is composed of three major domains: the head, rod, and tail, arranged from the N- to C-terminus (Fig. 2g). The purified recombinant head domain expressed in *Escherichia coli* bound strongly to the AZM-conjugated beads, indicating that AZM binds directly to the N-terminal head domain of KRT18 (Fig. 2h). KRT18 is expressed in simple epithelial cells, and keratin filament networks are required for epithelial tissues to maintain their stiffness and integrity under mechanical stress [33]. We explored the effect of AZM on KRT18 networks to understand how AZM inhibits autophagy by interacting with KRT18. In untreated control cells, GFP-KRT18 forms keratin filaments and exhibits dynamic intracellular movements from the cell periphery toward the centre of the cell (Video 1) [34]. AZM treatment suppresses the dynamic movement of keratin, while keratin filaments became thicker than those in untreated cells (Fig. 2i, Video 2). Our findings indicate that AZM interacts directly with KRT18 and inhibits its movement in A549 cells.

### KRT18 knockdown leads to autophagosome and lysosome accumulation along with autophagy inhibition

**Fig. 3:**
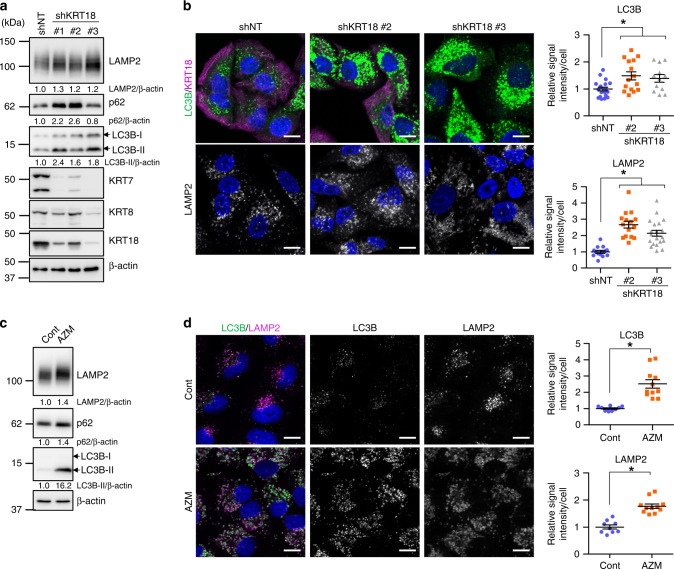
Keratin-18 knockdown leads to autophagosome and lysosome accumulation while inhibiting autophagy. **a** Expression of KRT7/8/18, LAMP2, p62, and LC3B in A549 cells expressing *shNT* or *shKRT18#1–3*, assessed using western blotting. **b** Immunofluorescence staining of LC3B, KRT18, and LAMP2 performed on A549 cells expressing *shNT*, *shKRT18#2*, or *shKRT18#3*. LC3B and LAMP2 are shown in green, while KRT18 is shown in magenta. Scale = 10 μm. Signal intensity of LC3B and LAMP2 was assessed and summarised in right. *p < 0.05 vs. shNT. **c** Expression of LAMP2, p62, and LC3B in control or AZM-treated (50 μM, 24 h) A549 cells, assessed using western blotting. **d** Immunofluorescence staining of LC3B and LAMP2 on AZM-treated A549 cells. Scale bar = 10 μm. Signal intensity of LC3B and LAMP2 was assessed and summarised in right. *p < 0.05 vs. control.

To determine whether KRT18 inhibition suppresses autophagy, we generated A549 cells expressing shRNA against *KRT18* (*shKRT18#1–3*). KRT18 forms a heterodimer with either KRT7 or KRT8 [35], both of which were identified as AZM-binding proteins (Fig. 2c). *KRT18* knockdown resulted in suppression of KRT7/8 expression, possibly due to the loss of a heterodimeric partner (Fig. 3a). LAMP2 and LC3B levels increased in *KRT18* knockdown cells, suggesting the accumulation of either autophagosomes, lysosomes, and/or autolysosomes (Fig. 3a). Additionally, p62 levels were higher in *shKRT18* cells, suggesting that *KRT18* knockdown inhibits autophagic flux (Fig. 3a). This hypothesis is supported by the flux assay, which shows reduced LC3-II and p62 accumulation after BafA1 treatment compared to control cells (shNT) (Fig. S3). Immunofluorescence staining exhibited an increase in LC3B-positive autophagosomes and LAMP2-positive lysosomes in KRT18 knockdown cells (Fig. 3b). This accumulation of LC3B and LAMP2 in AZM-treated cells was elucidated via western blotting and immunofluorescence staining (Fig. 3c, d). Collectively, our results suggest that AZM suppresses autophagy by interacting directly with KRT18 and disrupting the keratin network.

### AZM interacts with α/β-tubulin in non-epithelial cells

**Fig. 4:**
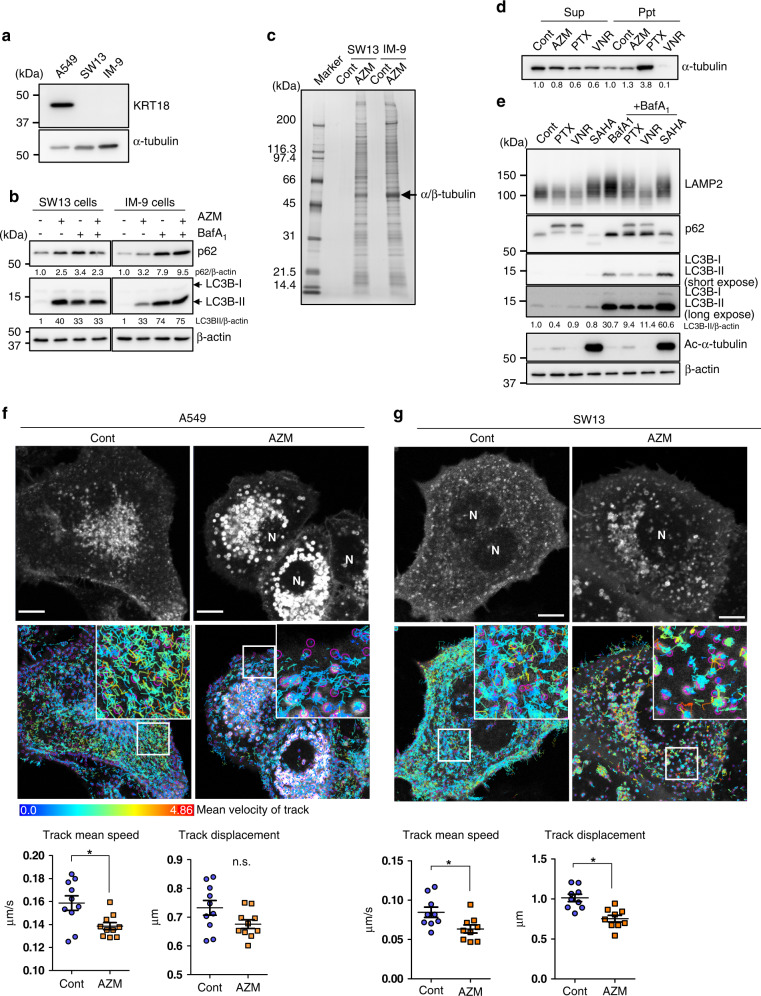
AZM interacted with α/β-tubulin in A549, SW13, and IM-9 cells. **a** KRT18 levels in A549, SW13, and IM-9 cells, determined via western blotting. α-tubulin was a loading control. **b** For autophagic flux assay, SW13 and IM-9 cells were treated with 50 μM AZM ± 10 nM BafA_1_ The band intensity of LC3B-II and p62 were standardised with β-actin and a relative value was shown below each panel. **c** Proteins from SW13 and IM-9 cells bound to control- and AZM-conjugated beads were visualised by silver staining. The major band was identified as α/β-tubulin by LC-MS/MS and is indicated with an arrow. **d** SW13 cells were treated with AZM, PTX (100 nM), or VNR (50 nM) for 24 h and separated into the Sup (containing depolymerised tubulin) and Ppt (containing polymerised tubulin) fractions and examined via western blotting for expression of α-tubulin. Relative band intensities were shown below the panel. **e** A549 cells treated with PTX, VNR, or SAHA with or without BafA_1_ for 24 h and assessed expression of LAMP2, p62 and LC3B by western blotting. The relative band intensity of LC3B-II was standardised with β-actin and shown. **f**, **g** AZM effects on lysosomal trafficking determined by LAMP1-EGFP-expressing A549 cells (**f**) or SW13 cells (**g**), 4 h after treatment with DMSO or AZM. Confocal microscopy images at time 0 (upper panels) and merged image with the mark of each vesicle with circle and path of each vesicle shown in coloured lines according to their mean velocity (lower panels). Scale = 10 μm. Vesicle displacement and mean velocity during a 200 s duration were calculated and summarised at the bottom. *n* = 10, 9. *p < 0.05. Time lapse videos used in **f**  are in Supplementary Videos 3 and 4.

KRT18 is expressed in simple epithelial cells but not in cells derived from the mesoderm, such as IM-9 and keratin-deficient SW13 cells [36] (Fig. 4a); however, AZM treatment inhibited autophagy in both IM-9 and SW13 cells (Fig. 4b), suggesting the presence of AZM targets other than KRT18 during autophagy inhibition. Thus, we attempted to identify AZM-binding proteins expressed by IM-9 and SW13 cells using AZM-conjugated beads. We identified α/β-tubulin as a major band, as noted with A549 cells (Figs. 2a, 4c arrow, Tables S3, S4).

We assessed effect of AZM on tubulin polymerisation. SW13 cells were treated with AZM, and tubulin was separated into non-polymerised (Sup) and polymerised (Ppt) fractions. Paclitaxel (PTX), which stabilises tubulin, and vinorelbine (VNR), which inhibits tubulin polymerisation, were used as controls. PTX increased α-tubulin level in Ppt, but VNR reduced it. We did not observe any effect of AZM on polymerisation (Fig. 4d). Microtubules act as a scaffold for intracellular vesicle trafficking, as autophagosomes and lysosomes are transported by microtubule-based dynein and kinesin motor proteins [37]. Tubulin inhibitors inhibit autophagy; PTX or VNR treatment inhibited autophagy, as shown by flux assay (Fig. 4e). Conversely, tubulin acetylation is reported to be involved in autophagosome formation under starvation conditions [38], and SAHA, which inhibits activity of the tubulin deacetylation enzyme HDAC6, increased autophagic flux (Fig. 4e). Therefore, we determined the effect of AZM on cytoplasmic vesicle trafficking by measuring lysosomal movement in A549 cells expressing LAMP1-EGFP. Tracking analysis revealed that AZM treatment reduces the rate of lysosome migration along microtubules (Fig. 4f, Video 3, 4), suggesting that AZM can alter autophagic flux by modulating intracellular vesicle trafficking. The same result was noted for SW13 cells, suggesting that the effect of AZM on tubulin is independent of KRT18 (Fig. 4g). However, tubulin inhibitors disrupted KRT18 intracellular dynamics (Fig. S4a, Video 5–7). Additionally, KRT18 knockdown suppressed tubulin acetylation in A549 cells (Fig. S4b, c). Although we did not observe any change in α-tubulin acetylation in response to AZM treatment (Fig. S4d), these data indicate an interaction between KRT18 and microtubules. Thus, we concluded that AZM inhibits autophagic flux via interaction with these cytoskeletal proteins.

### AZM does not perturb autolysosome formation

**Fig. 5:**
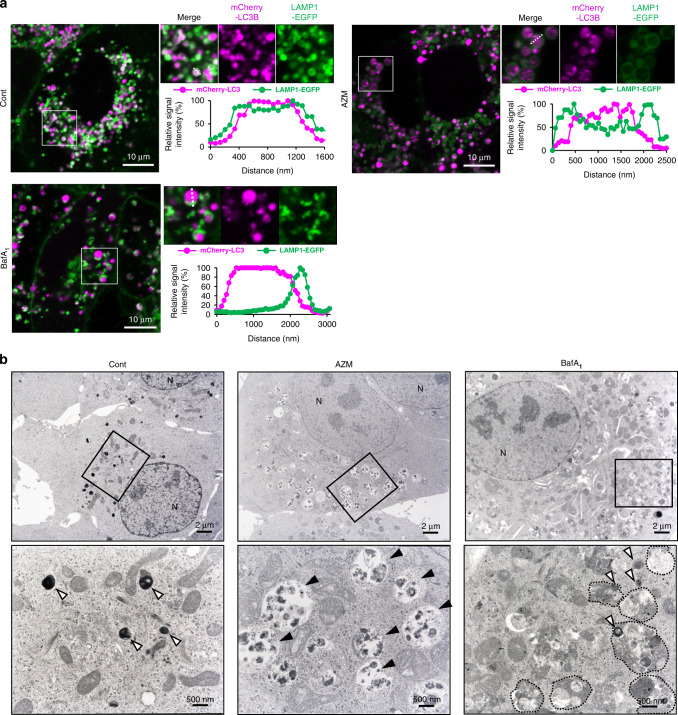
Effects of AZM on autolysosome formation and lysosomal function. **a** A549 cells expressing mCherry-LC3 and LAMP1-EGFP were treated with DMSO, 50 μM AZM, or 10 nM BafA_1_ for 24 h and monitored via confocal microcopy. Enlarged view in squares are shown on the right. Scale = 10 μm. Signal intensity of mCherry-LC3 and LAMP1-EGFP on the dashed line was calculated and summarised. **b** A549 cells were treated with DMSO, 50 μM AZM, or 10 nM BafA_1_ for 24 h and intracellular components were observed via TEM. Higher magnification view of boxed areas is shown on bottom. Scale = 2 μm for upper panels, and scale = 500 nm for lower panels. White arrowheads indicate lysosomes, while black arrowheads indicate autolysosomes.

We aimed to determine which steps of the autophagic process were affected by AZM. Immunofluorescence staining showed the accumulation of large, round LAMP2-positive vesicles in AZM-treated A549 cell cytoplasm, most of which contained LC3B-positive materials (Fig. S5), indicating the accumulation of enlarged autolysosomes. Similarly, the accumulation of autolysosomes was observed in mCherry-LC3B and LAMP1-EGFP-expressing A549 cells via confocal microscopy (Fig. 5a). In contrast, in BafA_1_-treated cells, most LAMP1-EGFP signals coincided with mCherry-LC3, but vesicle formation was perturbed (Fig. 5a). A similar result was obtained via immunofluorescence staining (Fig. S5), indicating impairment of autolysosome formation by BafA_1_ [28]. Furthermore, during transmission electron microscopy (TEM), we observed an increased number of enlarged autolysosomes engulfing incompletely digested material in AZM-treated cells (Fig. 5b). In BafA_1_-treated cells, we observed many deformed lysosomes adjacent to autophagosomes containing intact materials, probably due to the lack of fusion with lysosomes (Fig. 5b). These findings indicate that AZM treatment results in the accumulation of enlarged autolysosomes containing undigested debris. Unlike BafA_1_, AZM does not block autophagosome-lysosome fusion, but AZM appears to inhibit subsequent autolysosomal digestion.

### AZM inhibits lysosomal maturation and proteolysis

**Fig. 6:**
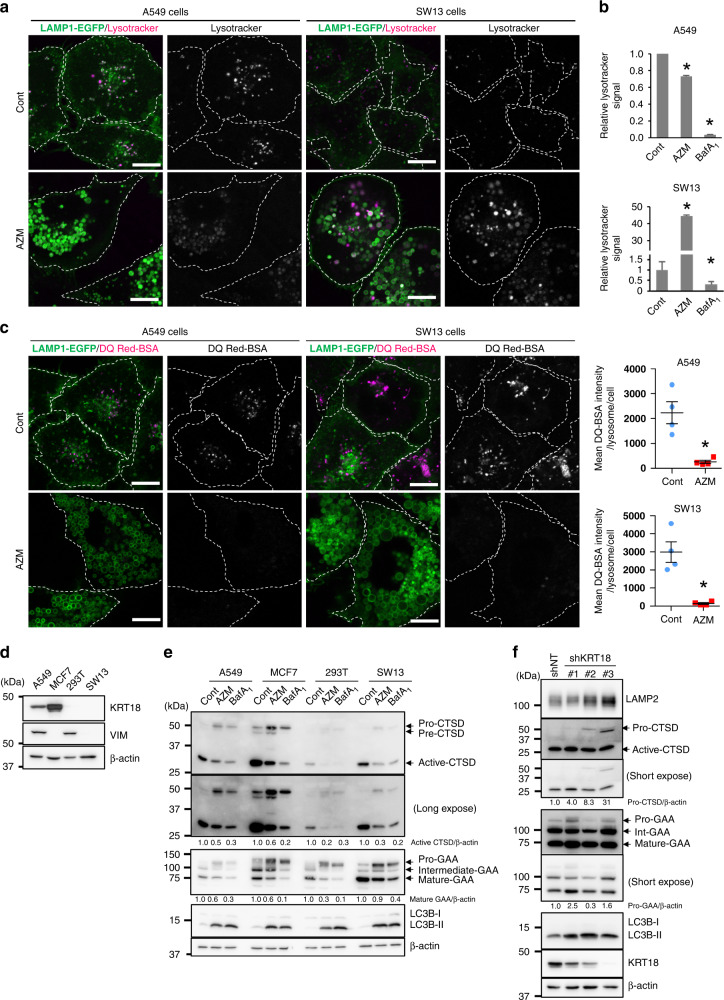
AZM disrupts lysosomal enzyme maturation independent of lysosomal pH. **a** A549 cells or SW13 cells expressing LAMP1-EGFP were treated with either DMSO or 50 μM AZM for 24 h and stained with LysoTracker Red, observed with confocal microscopy. Scale = 100 μm. **b** A549 cells or SW13 cells were treated with either DMSO or 50 μM AZM for 24 h and stained with LysoTracker Red; the signal intensity was assessed by flow cytometry. *n* = 3, bar = mean ± SD, *p < 0.05. **c** A549 or SW13 cells expressing LAMP1-EGFP were treated with DMSO or 50 μM AZM for 24 h and incubated with DQ Red BSA for 5 h in the presence of DMSO or AZM, observed with confocal microscopy. Scale = 100 μm. Signal intensity of DQ Red BSA in each cell, assessed and summarised on right. *n* = 4, bar = mean ± SD, *p < 0.05. **d** Expression of KRT18 and VIM in A549, MCF7, 293 T, and SW13 cells was assessed using western blotting. **e** Each of the indicated cells were treated with AZM (50 μM, 24 h) or BafA_1_ (10 nM, 24 h) and expression of CTSD, GAA, and LC3 was assessed via western blotting. The band intensity of active CTSD and mature GAA were standardised with β-actin and a relative value was shown below each panel. **f** Expression of KRT18, LAMP2, CTSD, GAA, and LC3B in A549 cells expressing *shNT* or *shKRT18#1–3* was assessed via western blotting. The band intensity of pro-CTSD and pro-GAA were standardised with β-actin and a relative value was shown below each panel.

We investigated lysosomal function following drug treatment to determine how AZM inhibits digestion of autolysosomal contents. As acidification is critical for lysosomal hydrolytic enzyme activity [39], A549 and SW13 cells were stained with LysoTracker Red to track lysosome acidity. Although AZM treatment weakened the signals slightly compared with control cells in A549, the signal increased in SW13 cells (Fig. 6a). The same result was noted for flow cytometry; BafA_1_-treatment exhibited inhibition of lysosomal acidification in both cell lines (Fig. 6b). We also used a mCherry-GFP-LC3 probe; GFP fluorescence is quenched at low pH that results from fusion with acidic lysosomes [40]. Treating A549 cells with AZM increased the number of GFP^+^/mCherry^+^ signals, indicating autolysosomes to not be acidified (Fig. S6). However, in SW13 cells, AZM treatment increased the abundance of GFP^-^/mCherry^+^ large vesicles, indicating an acidic environment of autolysosomes (Fig. S6). These results demonstrated that AZM-induced inhibition of lysosomal acidification is cell-type dependent.

Next, we determined lysosomal proteolytic activity by introducing DQ Red BSA into A549 or SW13 cells expressing LAMP1-EGFP. DQ Red BSA was incorporated into cells via endocytosis and delivered to lysosomes. Before proteolysis, fluorescence of DQ Red BSA was quenched, but once DQ Red BSA was exposed to proteolysis, it showed red fluorescence. We detected proteolytic activity in control cells 6 h after pulse-administration of DQ Red BSA. However, in the presence of AZM, proteolytic activity was almost undetectable (Fig. 6c). Endocytosis assessment using Dextran-Alexa488 indicated that AZM did not disrupt the endocytosis in A549 and SW13 cells (Fig. S7). These results demonstrated that AZM suppresses hydrolytic enzymatic activity in lysosomes, regardless of lysosomal acidity, and support the TEM findings showing the cytosolic accumulation of enlarged autolysosomes containing undigested debris (Fig. 5b). For further confirmation, we assessed the maturation of hydrolytic enzymes cathepsin D (CTSD), and α-glucosidase (GAA) via western blotting in KRT18-expressing A549 and MCF7 cells and KRT18-non-expressing 293 T and SW13 cells (Fig. 6d). CTSD and GAA mature via cleavage during lysosomal maturation [41, 42]. Notably, similar to A549 and SW13 cells, MCF7 cells showed a decrease in LysoTracker Red signals, whereas 293 T cells showed an increase in LysoTracker Red signals, in response to AZM treatment (Fig. S8). Since lysosomal acidity is an important condition for enzymatic maturation, BafA_1_ is used as a control to suppress lysosomal acidification (Fig. 6b, S8). In all cells, BafA_1_ treatment inhibited the maturation of CTSD and GAA, as indicated by repression of the active form of CTSD and mature-GAA (Fig. 6e). Similarly, AZM treatment decreased levels of the mature forms of CTSD and GAA, suggesting that AZM suppresses lysosomal enzymatic maturation, regardless of whether it inhibits lysosomal acidification. Finally, we assessed the involvement of KRT18 in maturation of CTSD and GAA. In *KRT18* knockdown cells, the levels of proforms of these enzymes increased slightly without an apparent decrease in the levels of mature forms of these enzymes (Fig. 6f), suggesting that KRT18 is involved in lysosomal maturation.

## Discussion

In this study, we elucidated that AZM specifically interacts with keratin-18 and α/β-tubulin, in addition to disturbing their cytoskeletal dynamics. Thus, we concluded that these cytoskeletal proteins are molecular targets of the autophagy inhibitory effect of AZM via suppression of trafficking of lysosomes and inhibition of lysosomal functional maturation. Since BafA_1_, a well-known autophagy inhibitor, has been reported to target both V-ATPase-dependent acidification and Ca-P60A/SERCA-dependent autophagosome-lysosome fusion independently for autophagy inhibition [28], it is not surprising that there are two targets of AZM for autophagy inhibition. Indeed, we have not obtained any data indicating a correlation between KRT18 expression level and AZM-induced growth inhibition in several cancer cell lines used in this study (data not shown). We have demonstrated that *KRT18* knockdown inhibits autophagic flux and partially suppresses lysosomal enzymatic maturation. Additionally, lysosomal trafficking on the microtubules was significantly repressed in the presence of AZM, which explains the autophagy inhibition in cells derived from mesoderm with no KRT18 expression. Since microtubules are critical for cellular physiology, including cell division, it was practically difficult to perform the experiments for α/β-tubulin knockdown. Thus, the involvement of α/β-tubulin in AZM-induced autophagy inhibition is still circumstantial. However, microtubules have a well-documented role in autophagy, which involves forming a scaffold for intracellular vesicle trafficking including autophagosomes and lysosomes [37].

In contrast to microtubules, the role of intermediate filaments, including keratin, in autophagic regulation is not well-documented. Our study showed that AZM disrupts intracellular dynamics of keratin filaments, thereby suppressing autophagy and accumulating autophagosomes, lysosomes, and autolysosomes (Figs. 2, 3, Video 2). Our findings indicate that KRT18 is a molecular target of AZM for autophagy inhibition (Figs. 3, 6). There are several reports supporting our findings, thereby suggesting keratin’s involvement in autophagy. In retinal pigment epithelium under paraquat-treated conditions, *KRT8*-knockdown suppressed autolysosome formation, but KRT8-overexpression promoted autolysosome formation [43]. In another study, *KRT8* knockdown suppressed autophagy and overcame chemoresistance of chordoma cells [44]. These results indicate the involvement of keratin filaments in autophagy.

Currently, there is no concrete evidence for the interaction between keratin and microtubules for autophagy inhibition; both were identified as the targets of AZM in this study. Notably, keratin knockdown reduced α-tubulin acetylation (Fig. S4b, c), along with autophagy inhibition, implying that keratin interacts with microtubules to regulate autophagy. Conversely, tubulin inhibitors disrupt keratin networks (Fig. S4a, Video 5–7). Although keratin filaments are known to move from the cell periphery to the cell centre (Video 1), the molecular mechanisms underlying these dynamics remain unclear [34]. Microtubule-based such retrograde transport is used for endosomal trafficking and is carried out by dynein, which is involved in autophagy regulation [37, 38]. It is crucial to understand the molecular mechanism underlying the interaction between microtubules and intermediate filaments during autophagy.

AZM possesses weakly basic lipophilic compounds, which accumulate in acidic organelles, including lysosomes. CQ is one of the well-known lysosomotropic compounds. These compounds increase lysosomal pH and disrupt lysosomal function because an acidic environment is critical for lysosomal hydrolytic enzyme activity [39]. Additionally, lysosomal enzymatic maturation was disturbed under high lysosomal pH because lysosomal enzymes are proteolytically processed and maturated by other lysosomal enzymes. However, in this study, although AZM inhibited lysosomal enzymatic maturation (Fig. 6e), it appeared to be independent of lysosomal pH; A549 and MCF7 cells showed a decreased LysoTracker Red signal, whereas it increased in SW13 and 293 T cells (Fig. 6a, Fig. S7). Additionally, this impaired enzyme maturation was partially observed in *KRT18* KD cells. Thus, further research should determine whether the lysosomal maturation inhibition is due to the repression of KRT18 movement or by the lysosomotropic compound itself.

Several ongoing clinical trials involving HCQ in cancer therapy involve the combination of HCQ with other anticancer drugs. Combination chemotherapy with HCQ has shown improved clinical outcomes in cases of glioblastoma and pancreatic ductal adenocarcinoma [45–47], but some trials did not show beneficial outcomes or sufficient benefit to warrant further study when compared with combination chemotherapies currently available [13, 48]. Throughout these trials, despite promising certain therapeutic effects, it has been difficult to demonstrate actual autophagy inhibition in response to HCQ in cancer patients. Additionally, some patients cannot receive an effective dose of HCQ due to its toxicity, which has led to an unsatisfactory outcome [2, 12, 13]. Thus, developing other effective and feasible autophagy inhibitors is an important clinical issue. AZM has been in daily clinical use for a long time; it has a long half-life (68 h), high tissue penetration, and 10–100 times higher concentration in human tissues compared with serum [49, 50]. Although AZM has a short measured half-life of 4 h in mice compared with humans [51], we still observed significant anti-tumour effects in mice following oral administration (Fig. 1). Furthermore, AZM showed less toxicity than HCQ in vitro (Fig. S1). These findings suggest the superiority of AZM for cancer therapy as an autophagy inhibitor. Genetically autophagy-deficient mice (*atg5* or *atg7* KO mice) are born normally but die within one day due to insufficient nutrition [52, 53]. In the case of induced whole-body *atg7* deletion in adult mice, most mutant mice die within 2–3 months due to infection and neurodegeneration [8]. BafA_1_ cannot be clinically used due to toxicity [54]. These data demonstrate excessive autophagy inhibition may damage the whole body in clinical application. Supporting this notion, a new autophagic inhibitor Lys05, a dimeric CQ, strongly inhibits autophagy but also causes Paneth cell dysfunction and intestinal toxicity in mice. This outcome is identical to that reported in humans and in mice carrying mutations in the autophagy gene *Atg16l1* [55]. Since AZM was less toxic to cultured cells than HCQ, and we have not observed any toxicity in orally-administrated mice at 100 μg/g/day; the risk of adverse events may be limited. We believe that the growth inhibitory effect and cell toxicity of AZM are dependent on how much the cancer cells rely on autophagy for their growth or survival, as we previously demonstrated [21, 31].

In this study, we found that keratin-7/8/18 and α/β-tubulin are novel targets of AZM for autophagy inhibition. Several questions remain unanswered; it is unknown how KRT7/8/18 regulates autophagy and what molecule(s) are involved in lysosomal trafficking and maturation after the interaction between AZM and these cytoskeletal proteins. However, the identification of molecular targets of AZM and assessment of its antitumor effect in an orally administrated xenograft mice model lays the foundation for further clinical application of AZM as an autophagy inhibitor in cancer therapy.

## Supplementary information

Supplementary information

Video 1

Video 2

Video 3

Video 4

Video 5

Video 6

Video 7

## Data Availability

The datasets used and/or analysed during the current study are available from the corresponding author on reasonable request.
