# Supplementary material for: Azithromycin, a potent autophagy inhibitor for cancer therapy, perturbs cytoskeletal protein dynamics
Source: Br J Cancer. 2023 Mar 4;128(10):1838–49. doi: 10.1038/s41416-023-02210-4 (PMC10147625; doi:10.1038/s41416-023-02210-4)
Supplement: Supplementary file 1 — Supplementary information [file 41416_2023_2210_MOESM1_ESM.pdf]

Supporting information

Azithromycin, a potent autophagy inhibitor for cancer therapy, perturbs cytoskeletal protein dynamics

Naoharu Takano[a*], Masaki Hiramoto[a], Yumiko Yamada[a], Hiroko Kokuba[b], Mayumi Tokuhisa[a], Hirotsugu Hino[a], and Keisuke Miyazawa[a*]

[a] Department of Biochemistry, Tokyo Medial University, Tokyo, Japan; [b] Laboratory of Electron Microscopy, Tokyo Medial University, Tokyo, Japan

List of Supporting Information

Supplementary figures:

Figure S1. Analysis of confluency, GFP, and RFP signals separately in A549 cells expressing GFP-LC3/RFP-LC3DG.

Figure S2. AZM-conjugated bead binding proteins identified from A549 cells.

Figure S3. Autophagy flux analysis with KRT18 knockdown A549 cells.

Figure S4. Tubulin disruption affected keratin distribution.

Figure S5. Effects of AZM on autolysosome formation.

Figure S6. Effect of AZM on autolysosomal acidification.

Figure S7. AZM does not disrupt endocytosis.

Figure S8. Effect of AZM on lysosomal acidification.

Supplementary Tables:

Table S1. Oligo DNA sequence used for knockout vector construction.

Table S2. List of identified AZM-beads binding proteins from A549 cells.

Table S3. List of identified AZM-beads binding proteins from SW13 cells.

Table S4. List of identified AZM-beads binding proteins from IM-9 cells.

Supplementary Videos:

Video 1. Time-lapse microscopy of A549 cells expressing GFP-KRT18.

Video 2. AZM suppresses dynamic intracellular movement of GFP-KRT18.

Video 3. Time-lapse microscopy of LAMP1-EGFP expressing A549 cells.

Video 4. AZM suppresses intracellular movement of LAMP1-EGFP.

Video 5. Time-lapse microscopy of A549 cells expressing GFP-KRT18.

Video 6. PTX suppresses dynamic intracellular movement of GFP-KRT18.

Video 7. VNR suppresses dynamic intracellular movement of GFP-KRT18.

Supplementary Material and Methods

References

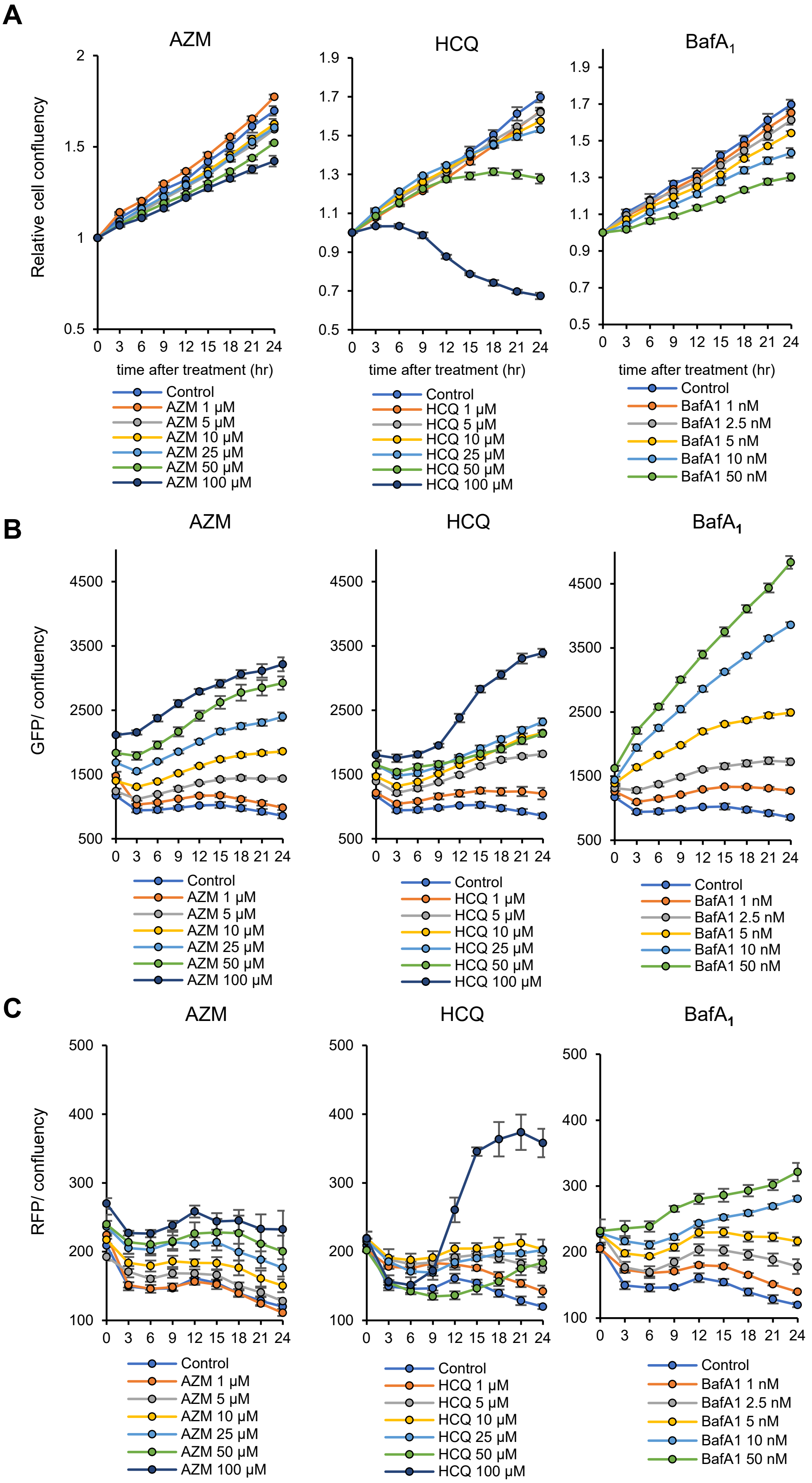

Figure S1. Analysis of confluency, GFP, and RFP signals separately in A549 cells expressing GFP-LC3/RFP-LC3ΔG. (A) Cell confluency was monitored simultaneously with fluorescence. Relative confluency at time 0 h was calculated and summarized. (B, C) GFP/RFP fluorescence signals were normalized to cell confluency and summarized. Signals obtained from the experiments shown in Fig. 1C. n = 4, bar = mean ± SE.

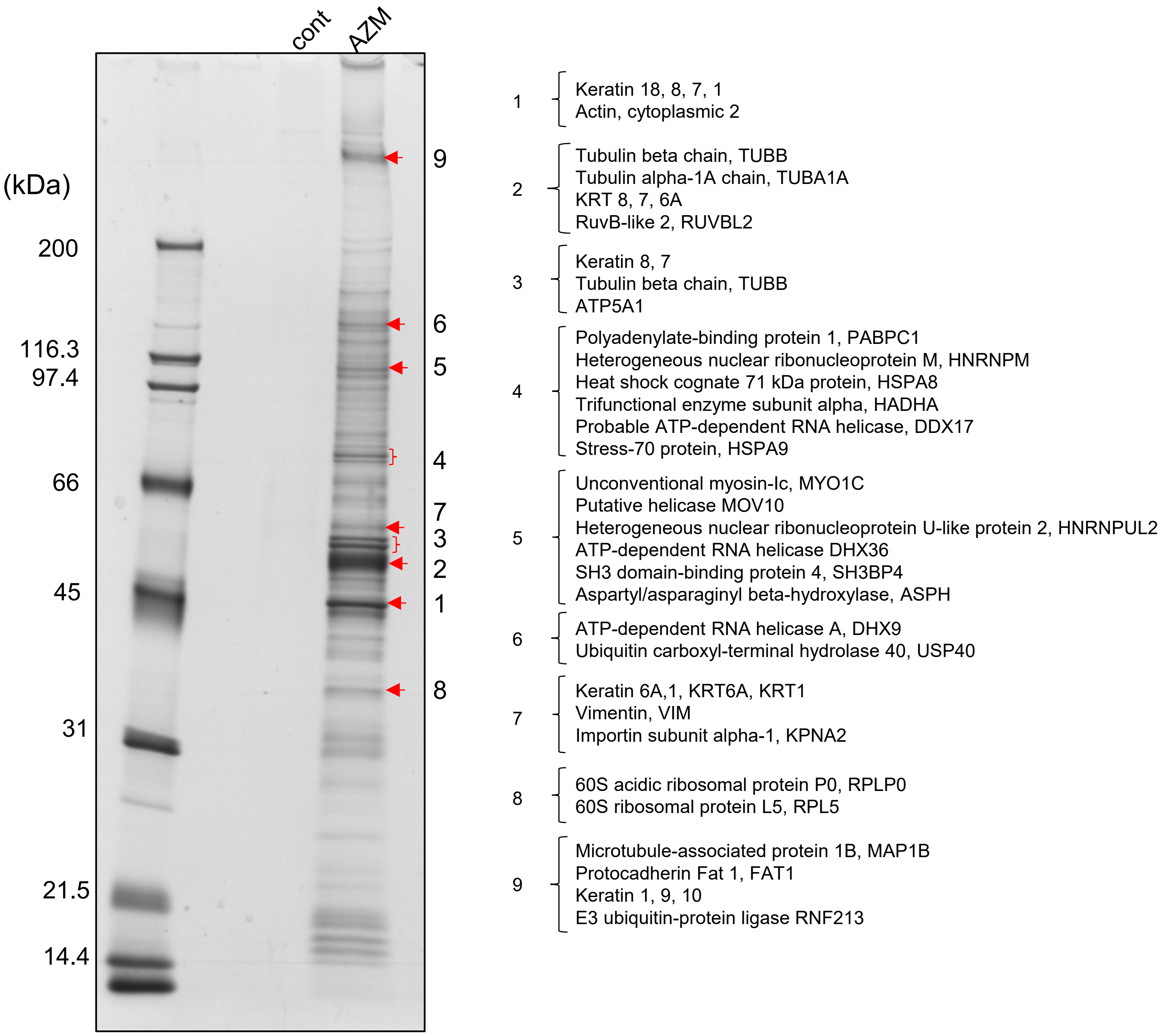

Figure S2. AZM-conjugated bead binding proteins identified from A549 cells. Proteins bound to AZM-conjugated and control beads were separated by SDS-PAGE and visualized using silver staining (same with Fig. 2C). Proteins identified via LC-MS/MS from indicated bands listed on the right.

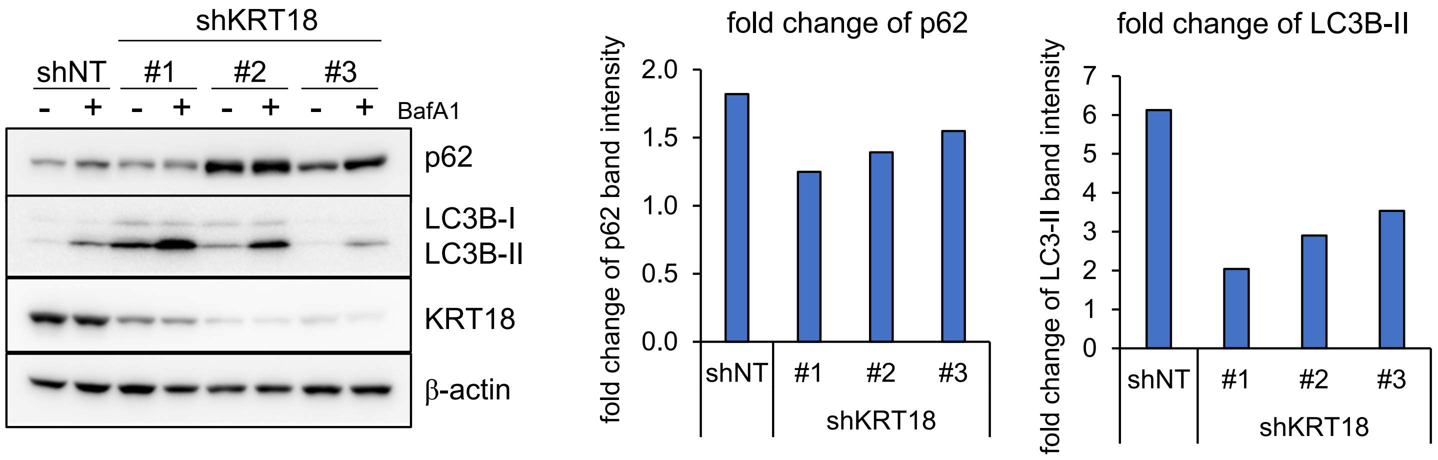

Figure S3. Autophagy flux analysis with KRT18 knockdown A549 cells. Control shNT and keratin knock downed shKRT18 A549 cells were treated with 10 nM $BafA_1$ for 4 h, and LC3 and p62 expression were assessed by western blotting. Band intensity of p62 or LC3-II were standardized with β–actin band intensity and compared between $BafA_1$-treated and -untreated samples.

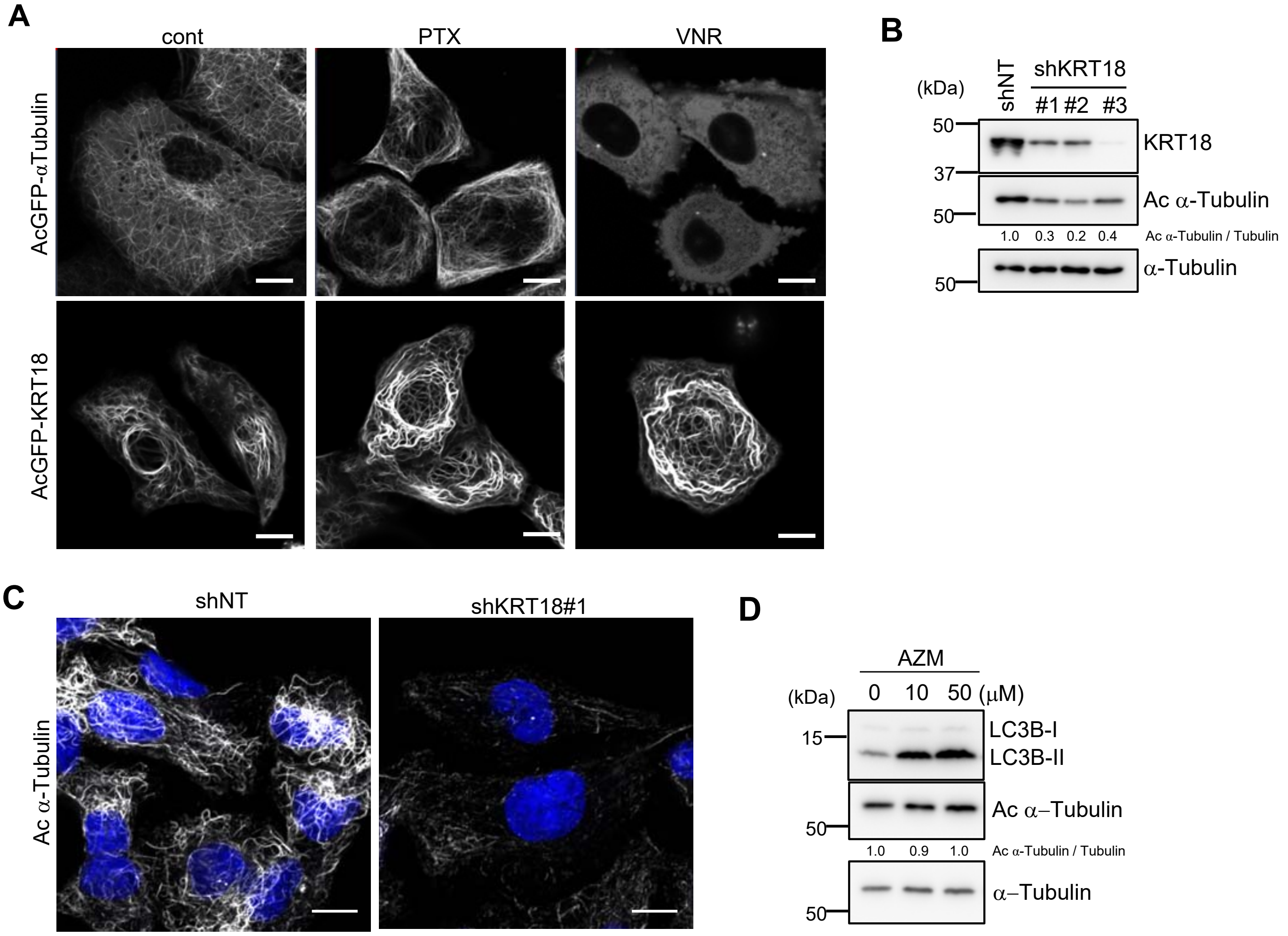

Figure S4. Tubulin disruption affected keratin distribution. (A) AcGFP-αTubulin-expressing A549 cells (upper panels) and AcGFP-KRT18-expressing A549 cells (lower panels) were treated with PTX (100 nM) or VNR (50 nM) for 24 h and observed using confocal microscopy. Scale = 10 μm. (B) Acetylated α-tubulin levels in KRT18 knockdown A549 cells, as determined via western blotting. (C) Immunofluorescence staining for acetylated α-tubulin (white). Nuclei were counterstained with DAPI (blue). Scale = 10 μm. (D) Acetylated α-tubulin levels in AZM-treated A549 cells, assessed via western blotting. A549 cells treated with AZM for 24 h at 10 or 50 μM.

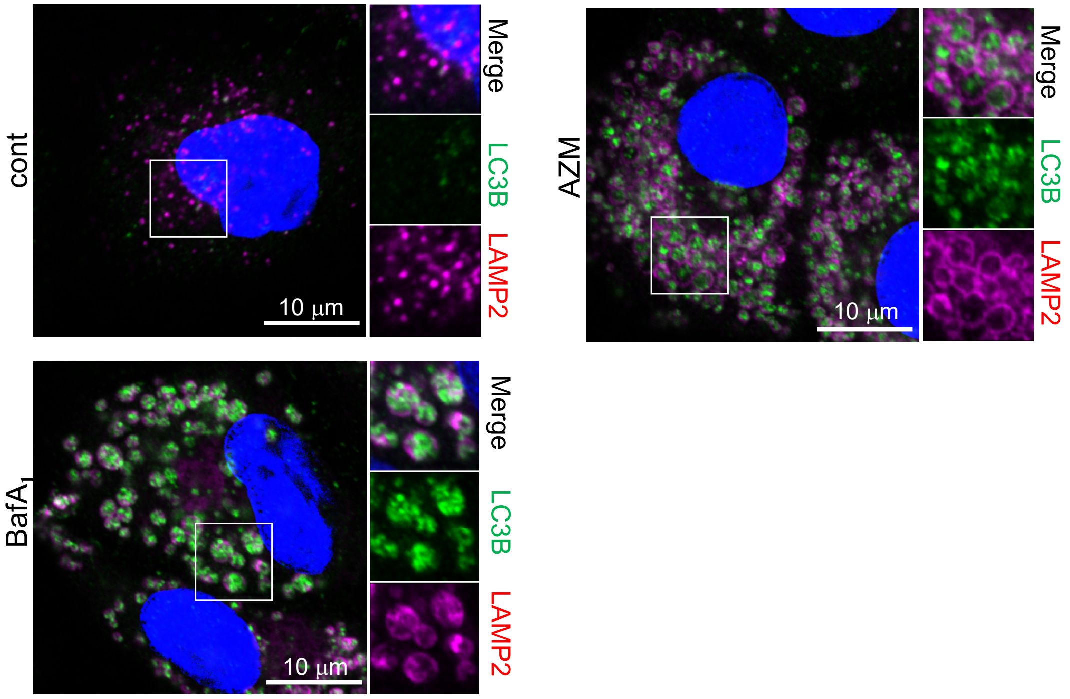

Figure S5. Effects of AZM on autolysosome formation. A549 cells were treated with DMSO, 50 μM AZM, or 10 nM BafA1 for 24 h before being immunostained for LC3 (Green) and LAMP2 (Magenta). Nuclei were counterstained with DAPI (blue). Boxed areas were enlarged in right panels. Scale = 10 μm

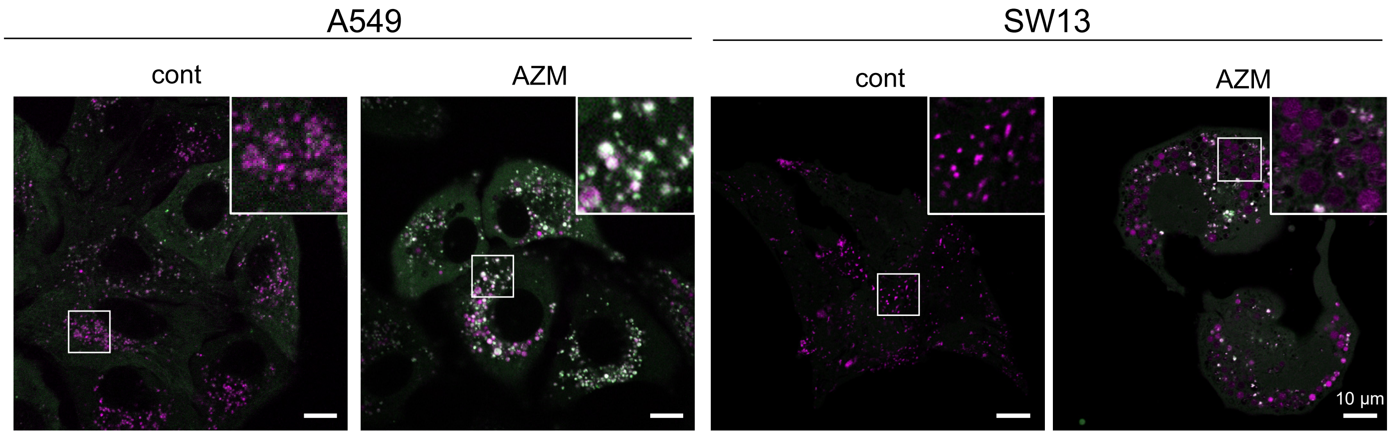

Figure S6. Effect of AZM on autolysosomal acidification. mCherry-EGFP-LC3-expressing A549 and SW13 cells were treated with 50 µM AZM for 24 h and observed via confocal microscopy. GFP signals (green) and mCherry signals (magenta). Scale = 10 µm.

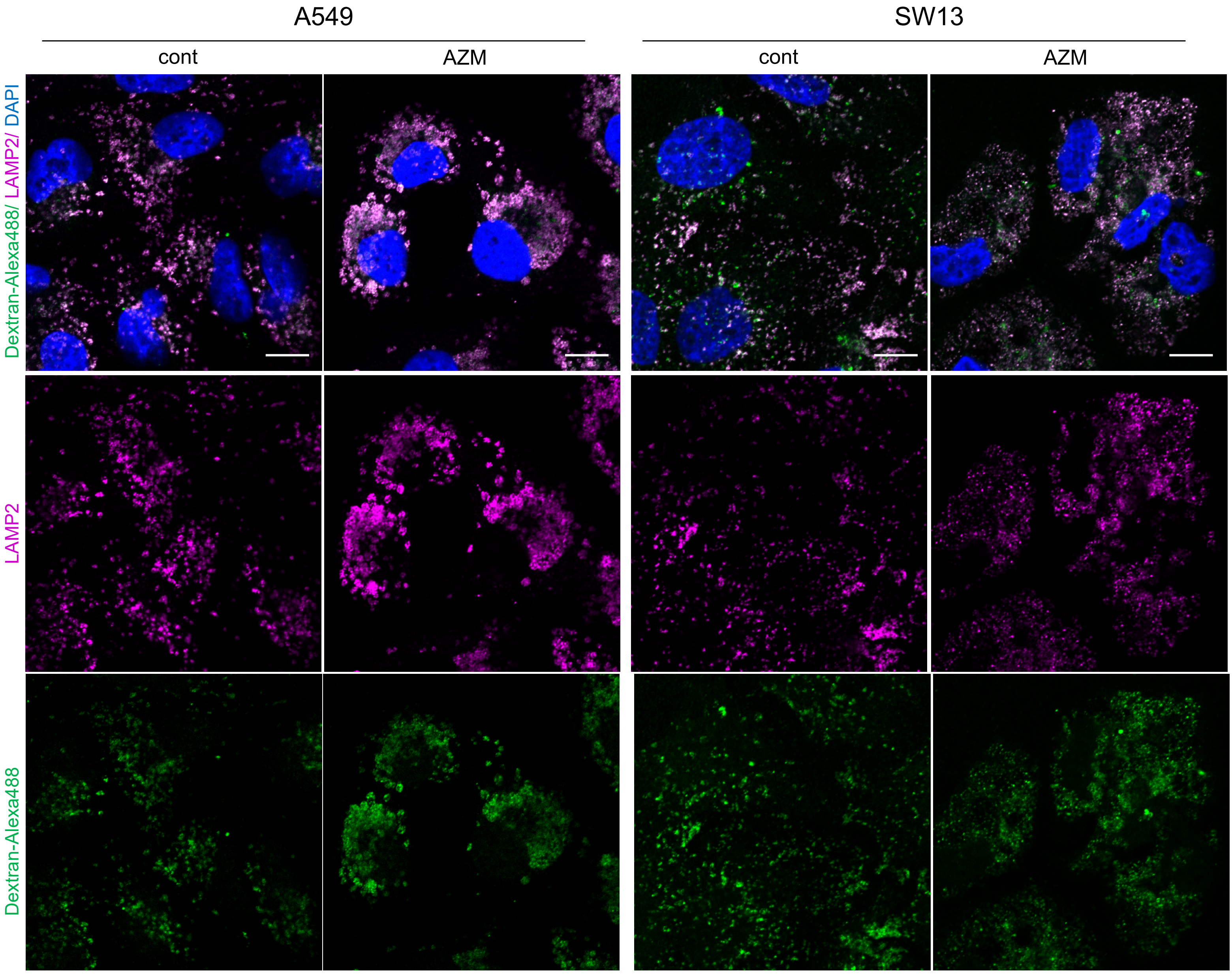

Figure S7. AZM does not disrupt endocytosis. A549 and SW13 cells were treated with 50 μM AZM for 24 h and incubated with dextran-Alexa488 for 6 h. Subsequently, cells were immunostained for LAMP2 and observed via confocal microscopy. Alexa488 signals, green; LAMP2 signals, magenta. Scale = 10 μm

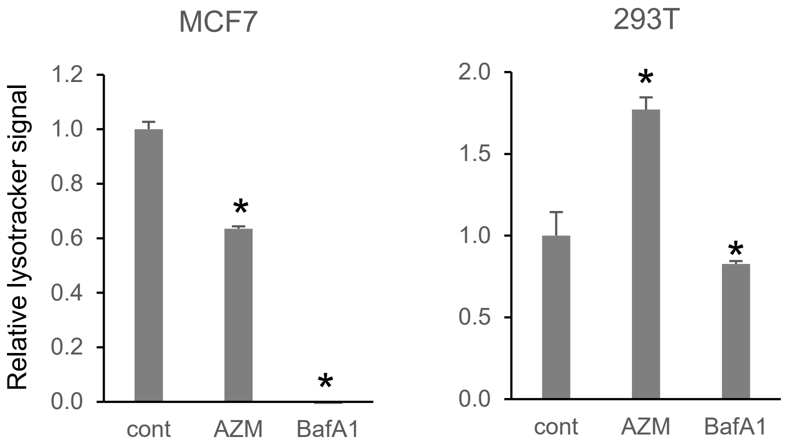

Figure S8. Effect of AZM on lysosomal acidification. MCF7 or 293T cells treated with DMSO, 50 μM AZM, or 10 nM BafA1 for 24 h and stained with lysotracker; the signal intensity was assessed using flow cytometry. n = 3, bar = mean ± SD, * $p < 0.05$.

Designation	Sequence
**DNA sequence for shRNA vector**	
shNT	5’-CCGGCAACAAGATGAAGAGCACCAACTCGAGTTGGTGCTCTTCATCTTGTTGTTTTTG-3’
shKRT18#1	5’- CCGGCAGATTGACAATGCCCGTCTTCTCGAGAAGACGGGCATTGTCAATCTGTTTTTG-3’
shKRT18#2	5’- CCGGCTTCATGAAGAAGAACCACGACTCGAGTCGTGGTTCTTCTTCATGAAGTTTTTG-3’
shKRT18#3	5’- CCGGGATGACACCAATATCACACGACTCGAGTCGTGTGATATTGGTGTCATCTTTTTG-3’

Table S1. shRNA sequences. Related to Methods. The DNA sequences used for shRNA vector construction are listed.

Unused	Total	% Cov (95)	Accession #	Name	Species	Peptides(95%)
127.92	127.92	85.6	sp|P05783|K1C18_HUMAN	Keratin, type I cytoskeletal 18 OS=Homo sapiens GN=KRT18 PE=1 SV=2	HUMAN	237
119	119	77.9	sp|P07437|TBB5_HUMAN	Tubulin beta chain OS=Homo sapiens GN=TUBB PE=1 SV=2	HUMAN	224
118.17	118.17	83.9	sp|P05787|K2C8_HUMAN	Keratin, type II cytoskeletal 8 OS=Homo sapiens GN=KRT8 PE=1 SV=7	HUMAN	213
104.22	104.22	74.9	sp|Q71U36|TBA1A_HUMAN	Tubulin alpha-1A chain OS=Homo sapiens GN=TUBA1A PE=1 SV=1	HUMAN	153
80.31	98.22	75.5	sp|P08729|K2C7_HUMAN	Keratin, type II cytoskeletal 7 OS=Homo sapiens GN=KRT7 PE=1 SV=5	HUMAN	140
29.51	80.83	61.7	sp|Q9BUF5|TBB6_HUMAN	Tubulin beta-6 chain OS=Homo sapiens GN=TUBB6 PE=1 SV=1	HUMAN	126
102.78	102.78	59	sp|P11940|PABP1_HUMAN	Polyadenylate-binding protein 1 OS=Homo sapiens GN=PABPC1 PE=1 SV=2	HUMAN	115
64.27	64.27	40.1	sp|P52272|HNRPM_HUMAN	Heterogeneous nuclear ribonucleoprotein M OS=Homo sapiens GN=HNRNPM PE=1 SV=3	HUMAN	65
91.61	91.61	16.7	sp|P46821|MAP1B_HUMAN	Microtubule-associated protein 1B OS=Homo sapiens GN=MAP1B PE=1 SV=2	HUMAN	52
73.72	73.72	51.4	sp|P02538|K2C6A_HUMAN	Keratin, type II cytoskeletal 6A OS=Homo sapiens GN=KRT6A PE=1 SV=3	HUMAN	47
57.54	57.54	32.3	sp|P04264|K2C1_HUMAN	Keratin, type II cytoskeletal 1 OS=Homo sapiens GN=KRT1 PE=1 SV=6	HUMAN	45
58.46	58.46	44.3	sp|P11142|HSP7C_HUMAN	Heat shock cognate 71 kDa protein OS=Homo sapiens GN=HSPA8 PE=1 SV=1	HUMAN	45
77.65	77.65	30.9	sp|O00159|MYO1C_HUMAN	Unconventional myosin-Ic OS=Homo sapiens GN=MYO1C PE=1 SV=4	HUMAN	45
56.41	56.44	49.2	sp|Q9Y230|RUVB2_HUMAN	RuvB-like 2 OS=Homo sapiens GN=RUVBL2 PE=1 SV=3	HUMAN	43
40.37	40.37	42	sp|P05388|RLA0_HUMAN	60S acidic ribosomal protein P0 OS=Homo sapiens GN=RPLP0 PE=1 SV=1	HUMAN	42
59.48	59.48	21	sp|Q08211|DHX9_HUMAN	ATP-dependent RNA helicase A OS=Homo sapiens GN=DHX9 PE=1 SV=4	HUMAN	41
64.65	64.65	30.2	sp|Q9HCE1|MOV10_HUMAN	Putative helicase MOV-10 OS=Homo sapiens GN=MOV10 PE=1 SV=2	HUMAN	38
40.01	40.01	29.2	sp|P35527|K1C9_HUMAN	Keratin, type I cytoskeletal 9 OS=Homo sapiens GN=KRT9 PE=1 SV=3	HUMAN	34
52.96	53.01	28.3	sp|Q9BUJ2|HNRL1_HUMAN	Heterogeneous nuclear ribonucleoprotein U-like protein 1 OS=Homo sapiens GN=HNRNPUL1 PE=1 SV=	HUMAN	34
53.18	53.18	30.4	sp|Q1KMD3|HNRL2_HUMAN	Heterogeneous nuclear ribonucleoprotein U-like protein 2 OS=Homo sapiens GN=HNRNPUL2 PE=1 SV=	HUMAN	34
34.17	34.17	35.4	sp|P52292|IMA1_HUMAN	Importin subunit alpha-1 OS=Homo sapiens GN=KPNA2 PE=1 SV=1	HUMAN	30
47.13	48.75	48.3	sp|P08670|VIME_HUMAN	Vimentin OS=Homo sapiens GN=VIM PE=1 SV=4	HUMAN	30
50.35	50.35	22.9	sp|Q9H2U1|DHX36_HUMAN	ATP-dependent RNA helicase DHX36 OS=Homo sapiens GN=DHX36 PE=1 SV=2	HUMAN	30
29.87	29.87	36.3	sp|P63261|ACTG_HUMAN	Actin, cytoplasmic 2 OS=Homo sapiens GN=ACTG1 PE=1 SV=1	HUMAN	27
34.89	34.89	39.4	sp|P46777|RL5_HUMAN	60S ribosomal protein L5 OS=Homo sapiens GN=RPL5 PE=1 SV=3	HUMAN	27
36	46	34.7	sp|P35908|K22E_HUMAN	Keratin, type II cytoskeletal 2 epidermal OS=Homo sapiens GN=KRT2 PE=1 SV=2	HUMAN	27
36.24	36.24	41.4	sp|P31943|HNRH1_HUMAN	Heterogeneous nuclear ribonucleoprotein H OS=Homo sapiens GN=HNRNPH1 PE=1 SV=4	HUMAN	27
37.47	39.69	30.8	sp|P13645|K1C10_HUMAN	Keratin, type I cytoskeletal 10 OS=Homo sapiens GN=KRT10 PE=1 SV=6	HUMAN	26
36.85	37.62	18.6	sp|Q96CW5|GCP3_HUMAN	Gamma-tubulin complex component 3 OS=Homo sapiens GN=TUBGCP3 PE=1 SV=2	HUMAN	25
31.12	35.25	25.8	sp|O00571|DDX3X_HUMAN	ATP-dependent RNA helicase DDX3X OS=Homo sapiens GN=DDX3X PE=1 SV=3	HUMAN	24
38.11	38.11	20	sp|Q9BSJ2|GCP2_HUMAN	Gamma-tubulin complex component 2 OS=Homo sapiens GN=TUBGCP2 PE=1 SV=2	HUMAN	24
40.47	40.47	20.5	sp|Q9P0V3|SH3B4_HUMAN	SH3 domain-binding protein 4 OS=Homo sapiens GN=SH3BP4 PE=1 SV=1	HUMAN	23
35.05	36.23	24.3	sp|P38646|GRP75_HUMAN	Stress-70 protein, mitochondrial OS=Homo sapiens GN=HSPA9 PE=1 SV=2	HUMAN	22
35.37	35.37	21.8	sp|Q12797|ASPH_HUMAN	Aspartyl/asparaginyl beta-hydroxylase OS=Homo sapiens GN=ASPH PE=1 SV=3	HUMAN	22
38.46	38.5	25.5	sp|Q92841|DDX17_HUMAN	Probable ATP-dependent RNA helicase DDX17 OS=Homo sapiens GN=DDX17 PE=1 SV=2	HUMAN	22
40.13	40.2	17.6	sp|P51530|DNA2_HUMAN	DNA replication ATP-dependent helicase/nuclease DNA2 OS=Homo sapiens GN=DNA2 PE=1 SV=3	HUMAN	22
40.62	40.62	14.9	sp|Q9NVE5|UBP40_HUMAN	Ubiquitin carboxyl-terminal hydrolase 40 OS=Homo sapiens GN=USP40 PE=1 SV=3	HUMAN	22
28.71	28.71	23.4	sp|Q9Y5X1|SNX9_HUMAN	Sorting nexin-9 OS=Homo sapiens GN=SNX9 PE=1 SV=1	HUMAN	21
39.28	39.28	27.4	sp|P40939|ECHA_HUMAN	Trifunctional enzyme subunit alpha, mitochondrial OS=Homo sapiens GN=HADHA PE=1 SV=2	HUMAN	21
27.28	27.7	30.4	sp|P63151|2ABA_HUMAN	Serine/threonine-protein phosphatase 2A 55 kDa regulatory subunit B alpha isoform OS=Homo sapiens	HUMAN	20
28.89	29.02	38.4	sp|P36578|RL4_HUMAN	60S ribosomal protein L4 OS=Homo sapiens GN=RPL4 PE=1 SV=5	HUMAN	20
21.11	21.11	17.3	sp|Q9NZI8|IF2B1_HUMAN	Insulin-like growth factor 2 mRNA-binding protein 1 OS=Homo sapiens GN=IGF2BP1 PE=1 SV=2	HUMAN	15
25.37	25.37	5	sp|P27708|PYR1_HUMAN	CAD protein OS=Homo sapiens GN=CAD PE=1 SV=3	HUMAN	13
20.78	21.04	19.5	sp|P25705|ATPA_HUMAN	ATP synthase subunit alpha, mitochondrial OS=Homo sapiens GN=ATP5A1 PE=1 SV=1	HUMAN	13
12.89	12.94	9.6	sp|Q08380|LG3BP_HUMAN	Galectin-3-binding protein OS=Homo sapiens GN=LGALS3BP PE=1 SV=1	HUMAN	7

Table S2. List of identified AZM-beads binding proteins from A549 cells

Unused	Total	%Cov(95)	Accession	Name	Species	Peptides(95%)
103.97	103.97	77.03	sp|P07437|TBB5_HUMAN	Tubulin beta chain OS=Homo sapiens GN=TUBB PE=1 SV=2	HUMAN	156
89.02	89.02	84.26	sp|P68363|TBA1B_HUMAN	Tubulin alpha-1B chain OS=Homo sapiens GN=TUBA1B PE=1 SV=1	HUMAN	94
79.02	79.02	53.7	sp|P52272|HNRPM_HUMAN	Heterogeneous nuclear ribonucleoprotein M OS=Homo sapiens GN=HNRNPM PE=	HUMAN	84
52.18	88.89	58.65	sp|P11940|PABP1_HUMAN	Polyadenylate-binding protein 1 OS=Homo sapiens GN=PABPC1 PE=1 SV=2	HUMAN	77
89.07	89.07	57.45	sp|Q13310|PABP4_HUMAN	Polyadenylate-binding protein 4 OS=Homo sapiens GN=PABPC4 PE=1 SV=1	HUMAN	74
104.99	104.99	24.27	sp|P27708|PYR1_HUMAN	CAD protein OS=Homo sapiens GN=CAD PE=1 SV=3	HUMAN	57
77.02	77.02	54.92	sp|Q9Y6M1|IF2B2_HUMAN	Insulin-like growth factor 2 mRNA-binding protein 2 OS=Homo sapiens GN=IGF2B	HUMAN	56
74.01	74.01	22.77	sp|Q9NYU2|UGGG1_HUMAN	UDP-glucose:glycoprotein glucosyltransferase 1 OS=Homo sapiens GN=UGGT1 PI	HUMAN	53
51.07	57.95	46.62	sp|Q9NZI8|IF2B1_HUMAN	Insulin-like growth factor 2 mRNA-binding protein 1 OS=Homo sapiens GN=IGF2B	HUMAN	45
42.95	58.31	47.32	sp|O00425|IF2B3_HUMAN	Insulin-like growth factor 2 mRNA-binding protein 3 OS=Homo sapiens GN=IGF2B	HUMAN	41
57.5	57.5	23.7	sp|Q08211|DHX9_HUMAN	ATP-dependent RNA helicase A OS=Homo sapiens GN=DHX9 PE=1 SV=4	HUMAN	36
46.01	46.01	42.98	sp|P13645|K1C10_HUMAN	Keratin, type I cytoskeletal 10 OS=Homo sapiens GN=KRT10 PE=1 SV=6	HUMAN	33
48.33	48.33	53.49	sp|P08779|K1C16_HUMAN	Keratin, type I cytoskeletal 16 OS=Homo sapiens GN=KRT16 PE=1 SV=4	HUMAN	32
62	62	33.9	sp|Q9HCE1|MOV10_HUMAN	Putative helicase MOV-10 OS=Homo sapiens GN=MOV10 PE=1 SV=2	HUMAN	32
35.65	35.65	62.15	sp|P63244|RACK1_HUMAN	Receptor of activated protein C kinase 1 OS=Homo sapiens GN=RACK1 PE=1 SV=	HUMAN	30
50.58	50.58	46.45	sp|P02538|K2C6A_HUMAN	Keratin, type II cytoskeletal 6A OS=Homo sapiens GN=KRT6A PE=1 SV=3	HUMAN	30
40.28	40.28	16.89	sp|P12107|COBA1_HUMAN	Collagen alpha-1(XI) chain OS=Homo sapiens GN=COL11A1 PE=1 SV=4	HUMAN	28
44.64	44.64	41.49	sp|P11142|HSP7C_HUMAN	Heat shock cognate 71 kDa protein OS=Homo sapiens GN=HSPA8 PE=1 SV=1	HUMAN	28
32.27	32.27	66.67	sp|P23396|RS3_HUMAN	40S ribosomal protein S3 OS=Homo sapiens GN=RPS3 PE=1 SV=2	HUMAN	26
45.79	45.79	31.8	sp|Q9NR30|DDX21_HUMAN	Nucleolar RNA helicase 2 OS=Homo sapiens GN=DDX21 PE=1 SV=5	HUMAN	26
41.59	41.62	26.75	sp|P07384|CAN1_HUMAN	Calpain-1 catalytic subunit OS=Homo sapiens GN=CAPN1 PE=1 SV=1	HUMAN	25
42.71	42.71	33.61	sp|Q92841|DDX17_HUMAN	Probable ATP-dependent RNA helicase DDX17 OS=Homo sapiens GN=DDX17 PE=	HUMAN	25
43.32	43.32	32.9	sp|P17844|DDX5_HUMAN	Probable ATP-dependent RNA helicase DDX5 OS=Homo sapiens GN=DDX5 PE=1	HUMAN	25
28.51	28.51	57.72	sp|P67809|YBOX1_HUMAN	Nuclease-sensitive element-binding protein 1 OS=Homo sapiens GN=YBX1 PE=1	HUMAN	24
34.92	34.94	45.56	sp|P18124|RL7_HUMAN	60S ribosomal protein L7 OS=Homo sapiens GN=RPL7 PE=1 SV=1	HUMAN	24
43.27	43.27	24.27	sp|Q12906|ILF3_HUMAN	Interleukin enhancer-binding factor 3 OS=Homo sapiens GN=ILF3 PE=1 SV=3	HUMAN	24
36.59	36.59	24.36	sp|Q1KMD3|HNRL2_HUMAN	Heterogeneous nuclear ribonucleoprotein U-like protein 2 OS=Homo sapiens GN=I	HUMAN	23
31.05	31.08	41.35	sp|P62424|RL7A_HUMAN	60S ribosomal protein L7a OS=Homo sapiens GN=RPL7A PE=1 SV=2	HUMAN	22
42	42	29.66	sp|P04264|K2C1_HUMAN	Keratin, type II cytoskeletal 1 OS=Homo sapiens GN=KRT1 PE=1 SV=6	HUMAN	22
33.19	39.89	36.62	sp|P35908|K22E_HUMAN	Keratin, type II cytoskeletal 2 epidermal OS=Homo sapiens GN=KRT2 PE=1 SV=2	HUMAN	21
34.01	34.01	52.23	sp|P13861|KAP2_HUMAN	cAMP-dependent protein kinase type II-alpha regulatory subunit OS=Homo sapien	HUMAN	21
35.55	37.55	33.71	sp|P35527|K1C9_HUMAN	Keratin, type I cytoskeletal 9 OS=Homo sapiens GN=KRT9 PE=1 SV=3	HUMAN	21
35.82	35.84	39.96	sp|Q9Y230|RUVB2_HUMAN	RuvB-like 2 OS=Homo sapiens GN=RUVBL2 PE=1 SV=3	HUMAN	21
26.05	26.05	44.1	sp|P31943|HNRH1_HUMAN	Heterogeneous nuclear ribonucleoprotein H OS=Homo sapiens GN=HNRNPH1 PE	HUMAN	20
26.09	26.09	31.55	sp|P05388|RLA0_HUMAN	60S acidic ribosomal protein P0 OS=Homo sapiens GN=RPLP0 PE=1 SV=1	HUMAN	20
30.62	30.62	45.88	sp|P46781|RS9_HUMAN	40S ribosomal protein S9 OS=Homo sapiens GN=RPS9 PE=1 SV=3	HUMAN	20
31.23	31.23	14.88	sp|P05997|CO5A2_HUMAN	Collagen alpha-2(V) chain OS=Homo sapiens GN=COL5A2 PE=1 SV=3	HUMAN	20
36.28	36.28	7.673	sp|Q5VU43|MYOME_HUMAN	Myomegalin OS=Homo sapiens GN=PDE4DIP PE=1 SV=1	HUMAN	20
36.79	36.79	53.24	sp|P63151|2ABA_HUMAN	Serine/threonine-protein phosphatase 2A 55 kDa regulatory subunit B alpha isofor	HUMAN	20
37.16	37.18	35.57	sp|Q9H6W3|NO66_HUMAN	Bifunctional lysine-specific demethylase and histidyl-hydroxylase NO66 OS=Homo	HUMAN	20
39.96	39.96	5.19	sp|Q99996|AKAP9_HUMAN	A-kinase anchor protein 9 OS=Homo sapiens GN=AKAP9 PE=1 SV=3	HUMAN	20

Table S3. List of identified AZM-beads binding proteins from SW13 cells

Unused	Total	%Cov(95)	Accession	Name	Species	Peptides(95%)
130.36	130.36	80.18	sp|P07437|TBB5_HUMAN	Tubulin beta chain OS=Homo sapiens GN=TUBB PE=1 SV=2	HUMAN	243
134.76	134.76	90.91	sp|Q71U36|TBA1A_HUMAN	Tubulin alpha-1A chain OS=Homo sapiens GN=TUBA1A PE=1 SV=1	HUMAN	187
188.14	188.14	43.01	sp|P27708|PYR1_HUMAN	CAD protein OS=Homo sapiens GN=CAD PE=1 SV=3	HUMAN	140
93.77	93.77	55.66	sp|P11940|PABP1_HUMAN	Polyadenylate-binding protein 1 OS=Homo sapiens GN=PABPC1 PE=1 SV=2	HUMAN	74
65.65	65.65	47.4	sp|P52272|HNRPM_HUMAN	Heterogeneous nuclear ribonucleoprotein M OS=Homo sapiens GN=HNRNPM PE=1	HUMAN	67
51.1	51.1	51.08	sp|P68104|EF1A1_HUMAN	Elongation factor 1-alpha 1 OS=Homo sapiens GN=EEF1A1 PE=1 SV=1	HUMAN	63
63.66	63.66	52.63	sp|P11142|HSP7C_HUMAN	Heat shock cognate 71 kDa protein OS=Homo sapiens GN=HSPA8 PE=1 SV=1	HUMAN	57
88.18	88.18	34.66	sp|Q9NYU2|UGGG1_HUMAN	UDP-glucose:glycoprotein glucosyltransferase 1 OS=Homo sapiens GN=UGGT1 PE=	HUMAN	53
75.6	75.6	36.58	sp|Q92900|RENT1_HUMAN	Regulator of nonsense transcripts 1 OS=Homo sapiens GN=UPF1 PE=1 SV=2	HUMAN	47
63.33	63.3	32.24	sp|Q12769|NU160_HUMAN	Nuclear pore complex protein Nup160 OS=Homo sapiens GN=NUP160 PE=1 SV=3	HUMAN	44
74.7	74.7	32.13	sp|Q08211|DHX9_HUMAN	ATP-dependent RNA helicase A OS=Homo sapiens GN=DHX9 PE=1 SV=4	HUMAN	44
25.28	56.11	39.29	sp|Q13310|PABP4_HUMAN	Polyadenylate-binding protein 4 OS=Homo sapiens GN=PABPC4 PE=1 SV=1	HUMAN	42
80.01	80.01	15.69	sp|Q92616|GCN1_HUMAN	eIF-2-alpha kinase activator GCN1 OS=Homo sapiens GN=GCN1 PE=1 SV=6	HUMAN	42
59.89	59.89	62.23	sp|P08670|VIME_HUMAN	Vimentin OS=Homo sapiens GN=VIM PE=1 SV=4	HUMAN	40
72.77	72.77	31.53	sp|P49790|NU153_HUMAN	Nuclear pore complex protein Nup153 OS=Homo sapiens GN=NUP153 PE=1 SV=2	HUMAN	40
57.16	57.16	49.53	sp|P04264|K2C1_HUMAN	Keratin, type II cytoskeletal 1 OS=Homo sapiens GN=KRT1 PE=1 SV=6	HUMAN	39
70.79	70.79	20.48	sp|Q92621|NU205_HUMAN	Nuclear pore complex protein Nup205 OS=Homo sapiens GN=NUP205 PE=1 SV=3	HUMAN	39
74.84	74.84	21.31	sp|Q92608|DOCK2_HUMAN	Dedicator of cytokinesis protein 2 OS=Homo sapiens GN=DOCK2 PE=1 SV=2	HUMAN	39
67.69	67.69	34.55	sp|Q6P2E9|EDC4_HUMAN	Enhancer of mRNA-decapping protein 4 OS=Homo sapiens GN=EDC4 PE=1 SV=1	HUMAN	38
43.44	43.54	41.07	sp|Q9NZI8|IF2B1_HUMAN	Insulin-like growth factor 2 mRNA-binding protein 1 OS=Homo sapiens GN=IGF2BP	HUMAN	37
65.89	65.89	30.28	sp|Q8WUM0|NU133_HUMAN	Nuclear pore complex protein Nup133 OS=Homo sapiens GN=NUP133 PE=1 SV=2	HUMAN	36
48.8	48.8	32.31	sp|Q14974|IMB1_HUMAN	Importin subunit beta-1 OS=Homo sapiens GN=KPNB1 PE=1 SV=2	HUMAN	34
56.51	56.51	32	sp|P57740|NU107_HUMAN	Nuclear pore complex protein Nup107 OS=Homo sapiens GN=NUP107 PE=1 SV=1	HUMAN	34
40.01	40.01	45.37	sp|P52292|IMA1_HUMAN	Importin subunit alpha-1 OS=Homo sapiens GN=KPNA2 PE=1 SV=1	HUMAN	33
56.69	56.69	31.14	sp|Q15477|SKIV2_HUMAN	Helicase SKI2W OS=Homo sapiens GN=SKIV2L PE=1 SV=3	HUMAN	33
58.65	58.65	45.4	sp|Q8N163|CCAR2_HUMAN	Cell cycle and apoptosis regulator protein 2 OS=Homo sapiens GN=CCAR2 PE=1 SV	HUMAN	33
31.64	31.64	64.35	sp|P63244|RACK1_HUMAN	Receptor of activated protein C kinase 1 OS=Homo sapiens GN=RACK1 PE=1 SV=3	HUMAN	32
49.14	49.14	52.92	sp|Q9Y230|RUVB2_HUMAN	RuvB-like 2 OS=Homo sapiens GN=RUVBL2 PE=1 SV=3	HUMAN	32
52.6	52.6	34.15	sp|Q8IYF3|TEX11_HUMAN	Testis-expressed protein 11 OS=Homo sapiens GN=TEX11 PE=1 SV=3	HUMAN	32
56.18	56.18	34.68	sp|Q8N1F7|NUP93_HUMAN	Nuclear pore complex protein Nup93 OS=Homo sapiens GN=NUP93 PE=1 SV=2	HUMAN	31
56.96	56.96	22.42	sp|Q99683|M3K5_HUMAN	Mitogen-activated protein kinase kinase kinase 5 OS=Homo sapiens GN=MAP3K5 F	HUMAN	31
60.59	60.59	39.47	sp|Q9BSJ2|GCP2_HUMAN	Gamma-tubulin complex component 2 OS=Homo sapiens GN=TUBGCP2 PE=1 SV=	HUMAN	31
53.15	53.15	30.33	sp|O95163|ELP1_HUMAN	Elongator complex protein 1 OS=Homo sapiens GN=IKBKAP PE=1 SV=3	HUMAN	30
60.03	60.03	34.03	sp|Q6PKG0|LARP1_HUMAN	La-related protein 1 OS=Homo sapiens GN=LARP1 PE=1 SV=2	HUMAN	30
52.21	52.2	29.01	sp|Q9HCE1|MOV10_HUMAN	Putative helicase MOV-10 OS=Homo sapiens GN=MOV10 PE=1 SV=2	HUMAN	29
37.24	37.24	46.77	sp|P31943|HNRH1_HUMAN	Heterogeneous nuclear ribonucleoprotein H OS=Homo sapiens GN=HNRNPH1 PE=	HUMAN	28
43.42	43.42	58.98	sp|P68363|TBA1B_HUMAN	Tubulin alpha-1B chain OS=Homo sapiens GN=TUBA1B PE=1 SV=1	HUMAN	28
36.35	36.35	51	sp|P23258|TBG1_HUMAN	Tubulin gamma-1 chain OS=Homo sapiens GN=TUBG1 PE=1 SV=2	HUMAN	27
43.61	43.61	36.16	sp|P17844|DDX5_HUMAN	Probable ATP-dependent RNA helicase DDX5 OS=Homo sapiens GN=DDX5 PE=1 S	HUMAN	27
45.69	45.69	55.18	sp|P08779|K1C16_HUMAN	Keratin, type I cytoskeletal 16 OS=Homo sapiens GN=KRT16 PE=1 SV=4	HUMAN	27
47.34	47.34	34.87	sp|Q9NR30|DDX21_HUMAN	Nucleolar RNA helicase 2 OS=Homo sapiens GN=DDX21 PE=1 SV=5	HUMAN	27
53	53	10.59	sp|P49327|FAS_HUMAN	Fatty acid synthase OS=Homo sapiens GN=FASN PE=1 SV=3	HUMAN	27
40.46	40.46	37.08	sp|P35527|K1C9_HUMAN	Keratin, type I cytoskeletal 9 OS=Homo sapiens GN=KRT9 PE=1 SV=3	HUMAN	26
46.24	46.24	20.94	sp|Q7L2E3|DHX30_HUMAN	Putative ATP-dependent RNA helicase DHX30 OS=Homo sapiens GN=DHX30 PE=1	HUMAN	26
50.18	50.18	35.72	sp|Q96CW5|GCP3_HUMAN	Gamma-tubulin complex component 3 OS=Homo sapiens GN=TUBGCP3 PE=1 SV=	HUMAN	26
31.6	31.6	39.95	sp|P39023|RL3_HUMAN	60S ribosomal protein L3 OS=Homo sapiens GN=RPL3 PE=1 SV=2	HUMAN	25
36	40.07	40.07	sp|O00425|IF2B3_HUMAN	Insulin-like growth factor 2 mRNA-binding protein 3 OS=Homo sapiens GN=IGF2BP	HUMAN	25
45.68	45.96	11.31	sp|Q13459|MYO9B_HUMAN	Unconventional myosin-IXb OS=Homo sapiens GN=MYO9B PE=1 SV=3	HUMAN	25
49.77	49.77	13.31	sp|O15027|SC16A_HUMAN	Protein transport protein Sec16A OS=Homo sapiens GN=SEC16A PE=1 SV=3	HUMAN	25
30.01	30.01	62.96	sp|P23396|RS3_HUMAN	40S ribosomal protein S3 OS=Homo sapiens GN=RPS3 PE=1 SV=2	HUMAN	24
34.96	34.96	35.65	sp|P30153|2AAA_HUMAN	Serine/threonine-protein phosphatase 2A 65 kDa regulatory subunit A alpha isoform	HUMAN	24
36.11	36.11	56.27	sp|P63261|ACTG_HUMAN	Actin, cytoplasmic 2 OS=Homo sapiens GN=ACTG1 PE=1 SV=1	HUMAN	24
40.36	40.36	68.21	sp|P67809|YBOX1_HUMAN	Nuclease-sensitive element-binding protein 1 OS=Homo sapiens GN=YBX1 PE=1 S	HUMAN	24
42.08	42.08	37.67	sp|P13645|K1C10_HUMAN	Keratin, type I cytoskeletal 10 OS=Homo sapiens GN=KRT10 PE=1 SV=6	HUMAN	24
45.98	45.98	17.18	sp|Q8TEQ6|GEMI5_HUMAN	Gem-associated protein 5 OS=Homo sapiens GN=GEMIN5 PE=1 SV=3	HUMAN	24
28.72	28.72	40.75	sp|P36578|RL4_HUMAN	60S ribosomal protein L4 OS=Homo sapiens GN=RPL4 PE=1 SV=5	HUMAN	23
28.82	28.82	43.85	sp|P05388|RLA0_HUMAN	60S acidic ribosomal protein P0 OS=Homo sapiens GN=RPLP0 PE=1 SV=1	HUMAN	23
38.57	38.57	25.29	sp|P13639|EF2_HUMAN	Elongation factor 2 OS=Homo sapiens GN=EEF2 PE=1 SV=4	HUMAN	23
34.01	34.01	43.37	sp|P52597|HNRPF_HUMAN	Heterogeneous nuclear ribonucleoprotein F OS=Homo sapiens GN=HNRNPF PE=1	HUMAN	22
35.23	35.26	24.89	sp|P49736|MCM2_HUMAN	DNA replication licensing factor MCM2 OS=Homo sapiens GN=MCM2 PE=1 SV=4	HUMAN	22
43.14	43.14	36.16	sp|P33993|MCM7_HUMAN	DNA replication licensing factor MCM7 OS=Homo sapiens GN=MCM7 PE=1 SV=4	HUMAN	22
33.22	40.01	34.74	sp|P35908|K22E_HUMAN	Keratin, type II cytoskeletal 2 epidermal OS=Homo sapiens GN=KRT2 PE=1 SV=2	HUMAN	21
36.71	36.74	37.01	sp|P27694|RFA1_HUMAN	Replication protein A 70 kDa DNA-binding subunit OS=Homo sapiens GN=RPA1 PE	HUMAN	21
36.8	36.8	32.93	sp|Q9BW27|NUP85_HUMAN	Nuclear pore complex protein Nup85 OS=Homo sapiens GN=NUP85 PE=1 SV=1	HUMAN	21
39.11	39.14	13.37	sp|P52948|NUP98_HUMAN	Nuclear pore complex protein Nup98-Nup96 OS=Homo sapiens GN=NUP98 PE=1 S	HUMAN	21
22.61	22.61	43.94	sp|P61247|RS3A_HUMAN	40S ribosomal protein S3a OS=Homo sapiens GN=RPS3A PE=1 SV=2	HUMAN	20
27.59	33.55	34.89	sp|Q9Y6M1|IF2B2_HUMAN	Insulin-like growth factor 2 mRNA-binding protein 2 OS=Homo sapiens GN=IGF2BP	HUMAN	20
29.1	29.21	52.47	sp|P62701|RS4X_HUMAN	40S ribosomal protein S4, X isoform OS=Homo sapiens GN=RPS4X PE=1 SV=2	HUMAN	20
30.32	30.37	51.61	sp|P18124|RL7_HUMAN	60S ribosomal protein L7 OS=Homo sapiens GN=RPL7 PE=1 SV=1	HUMAN	20
30.39	30.39	20.32	sp|Q8IWC1|MA7D3_HUMAN	MAP7 domain-containing protein 3 OS=Homo sapiens GN=MAP7D3 PE=1 SV=2	HUMAN	20
32.11	32.14	24.76	sp|Q9UHI6|DDX20_HUMAN	Probable ATP-dependent RNA helicase DDX20 OS=Homo sapiens GN=DDX20 PE=1	HUMAN	20
34.52	34.52	62.37	sp|P08865|RSSA_HUMAN	40S ribosomal protein SA OS=Homo sapiens GN=RPSA PE=1 SV=4	HUMAN	20
35.5	35.5	17.21	sp|P30876|RPB2_HUMAN	DNA-directed RNA polymerase II subunit RPB2 OS=Homo sapiens GN=POLR2B PE	HUMAN	20
35.82	35.82	8.538	sp|Q8N3C0|ASCC3_HUMAN	Activating signal cointegrator 1 complex subunit 3 OS=Homo sapiens GN=ASCC3 PE	HUMAN	20
37.96	38.12	18.64	sp|Q14697|GANAB_HUMAN	Neutral alpha-glucosidase AB OS=Homo sapiens GN=GANAB PE=1 SV=3	HUMAN	20
38.48	38.48	12.54	sp|Q00610|CLH1_HUMAN	Clathrin heavy chain 1 OS=Homo sapiens GN=CLTC PE=1 SV=5	HUMAN	20
39.82	39.82	13.04	sp|Q6PGP7|TTC37_HUMAN	Tetratricopeptide repeat protein 37 OS=Homo sapiens GN=TTC37 PE=1 SV=1	HUMAN	20
40.02	40.02	23.97	sp|Q8N8A2|ANR44_HUMAN	Serine/threonine-protein phosphatase 6 regulatory ankyrin repeat subunit B OS=Ho	HUMAN	20

Table S4. List of identified AZM-beads binding proteins from IM-9 cells

Video 1. Time-lapse microscopy of A549 cells expressing GFP-KRT18. Time-lapse images of A549 cells expressing GFP-KRT18 were obtained 12 h after DMSO treatment using confocal microscopy every 5 min for 12 h. Four different fields of view are shown sequentially.

Video 2. AZM suppresses dynamic intracellular movement of GFP-KRT18. Time lapse images of A549 cells expressing GFP-KRT18 were obtained 12 h after 50 μM AZM treatment with confocal microscopy every 5 min for 12 h. Four different fields of view are shown in sequence.

Video 3. Time-lapse microscopy of LAMP1-EGFP expressing A549 cells. Time lapse images of A549 cells expressing LAMP1-EGFP were obtained 4 h after DMSO treatment using confocal microscopy every 2 s for 200 s.

Video 4. AZM suppresses intracellular movement of LAMP1-EGFP. Time lapse images of A549 cells expressing LAMP1-EGFP were obtained 4 h after 50 μM AZM treatment with confocal microscopy every 2 s for 200 s.

Video 5. Time-lapse microscopy of A549 cells expressing GFP-KRT18. Time-lapse images of A549 cells expressing GFP-KRT18 were obtained 24 h after DMSO treatment using confocal microscopy every 5 min for 3 h.

Video 6. PTX suppresses dynamic intracellular movement of GFP-KRT18. Time-lapse images of A549 cells expressing GFP-KRT18 were obtained 24 h after PTX treatment using confocal microscopy every 5 min for 3 h.

Video 7. VNR suppresses dynamic intracellular movement of GFP-KRT18. Time-lapse images of A549 cells expressing GFP-KRT18 were obtained 24 h after VNR treatment using confocal microscopy every 5 min for 3 h.

Supplementary Materials and Methods

Cell culture. A549, MCF7, and IM-9 cell lines were obtained from ATCC. SW13 was obtained from JCRB Cell Bank. A549 and MCF7 cells were cultured in Dulbecco's Modified Eagle's medium (DMEM). IM-9 cells were cultured in RPMI1640, supplemented with 10% fetal bovine serum (FBS) and penicillin/streptomycin in a humidified 5% CO_2 incubator at 37°C. SW13 cells were cultured in Leibovitz's L-15 medium supplemented with 10% FBS and penicillin/streptomycin in a humidified incubator at 37°C. Mycoplasma contamination was routinely tested using the e-Myco Mycoplasma PCR Detection kit (iNtRON Biotechnology, # 25235). A549 cells expressing mCherry-GFP-LC3 were generated as previously described.[1]

Western blotting. Whole-cell lysates were prepared by lysing harvested cells in 1x RIPA buffer (Nacalai Tesque #08714-04) containing a protease inhibitor cocktail (Nacalai Tesque #25955-11) and phosphatase inhibitor cocktail (Nacalai Tesque #07575-51). Cell lysates were separated using SDS-PAGE and transferred by electroblotting on to a PVDF membrane. To detect unglycosylated LAMP2a, A549 cell lysates were treated with or without PNGaseF (New England BioLabs #P0704) and analyzed via western blotting. Western blotting was performed with the following antibodies obtained from Novus

Biologicals, Santa Cruz Biotechnology, Thermo Fisher Scientific, Sigma Millipore, BioLegend, Cell Signaling Technology, R&D systems, and Wako Pure Chemical Industries: anti-LC3B (Novus #NB600-1384), anti-p62 (Santa Cruz #sc-28359), anti-β-actin (Santa Cruz #sc-47778), anti-LAMP2 (Santa Cruz #sc-18822), anti-FLAG (Sigma #F1804), anti-GAA (R&D system #2489C), anti-CTSD (CST #2284), anti-KRT7 (CST #4465S), anti-KRT8 (abcam #ab9023), anti-KRT18 (BioLegend #628402), anti-α-tubulin (CST #3873), anti-acetyl-α-tubulin (Santa Cruz #sc-23950), anti-β-tubulin (CST #2146S), anti-VCP (BioLegend #636801), anti-VIM (Santa Cruz #sc-6260), anti-GST (Wako #013-21851), and anti-pp62 (CST #95697S).

Immunofluorescence staining. For immunofluorescence staining, cells were fixed in ice cold methanol at -20°C. To detect Ac-αTubulin, cells were washed with warm PBS and kept at 37°C until fixation. After blocking with 10% normal goat serum in TBST, cells were incubated with primary antibody (anti-LAMP2: Santa Cruz #sc-18822, anti-LC3B: CST #2775S, anti-Ac-α-tubulin: Santa Cruz #sc-23950, anti-KRT18: BioLegend #628402) diluted in blocking reagent for 16 h at 4°C. Primary antibodies were detected with either anti-mouse IgG or anti-rabbit IgG antibodies conjugated to either Alexa Fluor 488 or Alexa Fluor 555 (Invitrogen). To visualize acidic organelles, A549 cells were

stained with LysoTracker Red DND-99 (50 nM, Thermo #L7528) for 1 h, washed with PBS twice, and fixed with 2% paraformaldehyde for 10 min at RT. Nuclei were stained with 4',6-diamidino-2-phenylindole (DAPI). To analyze endocytosis, AZM-treated A549 cells or SW13 cells were incubated with Dextran Alexa Fluor 488 10,000 MW (Thermo #D22910) (50 μg/μL) for 5 h in the presence of AZM. Subsequently, cells were washed with PBS and fixed with 4% PFA for 10 min at RT. LAMP2 proteins were immunostained with anti-LAMP2 antibody, and nuclei were stained with DAPI. Fluorescence signals were observed by confocal microscopy (LSM700, Carl Zeiss). All images in each figure were acquired and processed in the same manner using ZEN 2012 software (Carl Zeiss).

Preparation of AZM-conjugated beads. To prepare AZM-conjugated beads, FG beads with carboxyl (-COOH) groups (#TAS8848N1140, Tamagawa seiki) and N',N'-di(desmethyl) Azithromycin (NH2-AZM) (Santa Cruz #sc-215514) were cross-linked with N-hydroxysuccinimide according to the manufacturer's instructions. First, 2.5 mg of FG-beads were washed with N',N'-dimethylformamide (DMF) 3 times and incubated with 0.2 M N-hydroxysuccinimide/0.2 M 1-ethyl-3-(3-dimethylaminopropyl)carbodiimide monohydrochloride in DMF for 2 h at RT. Subsequently, beads were washed five times with DMF and incubated with 2 mM NH2-

AZM/4 mM triethylamine in DMF with shaking for 70 min at RT. Next, beads were incubated with 2-aminoethanol for 2 h at RT to unmask unreacted COOH groups. Finally, beads were washed with 50% methanol three times and stored at 4°C. Control beads were prepared without NH2-AZM using the same procedure.

Isolation of AZM-conjugated beads binding proteins. A549, SW-13, and IM-9 cell lysates were prepared with binding buffer (20 mM HEPES-NaOH pH7.9/10% glycerol/0.1 M KCl/0.2 mM EDTA/1 mM $MgCl_2$/0.2 mM $CaCl_2$/0.1% NP-40/1 mM DTT/0.2 mM PMSF). AZM-conjugated and control beads were washed with binding buffer and incubated overnight with cell lysate at 4°C with shaking. Beads were subsequently washed with wash buffer (20 mM HEPES-NaOH pH7.9/10% glycerol/0.25 M KCl/0.2 mM EDTA/1 mM $MgCl_2$/0.2 mM $CaCl_2$/0.1% NP-40/1 mM DTT/0.2 mM PMSF) three times. To elute the binding proteins, beads were boiled with 2x SDS-PAGE sample buffer. Isolated proteins were separated using SDS-PAGE and visualized by silver staining. For LC-MS/MS analysis, gel was silver stained with SilverQuest™ Silver Staining Kit (Thermo Fisher Scientific #LC6070). Separated proteins were digested in-gel with trypsin and analyzed by LC-MS/MS (SCIEX TripleTOF 4600, SCIEX). Subsequent identification was done with ProteinPilot Software 5.0 (AB SCIEX, Framingham, MA).

Expression of recombinant Keratin-18. To express recombinant KRT18 protein, the head (residues 1–78), rod (residues 79–390), and tail (residues 391–430) domains of KRT18 were inserted into the pGEX6P1 expression vector (Cytiva) and transformed into *Escherichia coli* BL21(DE3)pLysS. To purify recombinant GST-KRT18 protein, IPTG-treated *E. coli* was first collected, freeze-thawed, and sonicated in PBS. Following sonication, the cell lysate was centrifuged, and the insoluble fraction was collected. To purify the inclusion bodies, the pellet was sonicated in 0.5% Triton-X100 before being centrifuged; this process was performed three times in total. The pellet was sonicated in 6 M urea/50 mM Tris-HCl pH 7.6/2 mM DTT and left overnight. The solubilized fraction was collected via centrifugation and used for dialysis with the following buffers and conditions: 8 M urea/25 mM Tris-HCl pH 7.6/10 mM β-mercaptoethanol at 4°C for 4 h, 2 M urea/5 mM Tris-HCl pH 7.6/5 mM β-mercaptoethanol at 4°C for 2 h, 5 mM Tris-HCl pH 7.6/5 mM β-mercaptoethanol at RT overnight as previously described.[2]

Tubulin separation. Polymerized and unpolymerized tubulin were separated as previously described.[3] Briefly, SW13 cells treated with various reagents were washed with warmed PBS at 37°C and harvested. Warmed hypotonic buffer (20 mM Tris-HCl pH 6.8/1% NP-

40/1 mM $MgCl_2$/2 mM EGTA) at 37°C, containing protease cocktail, was added to cell pellets and vortexed. Subsequently, cell lysates were centrifuged at 14 k rpm for 10 min. Supernatant was used as unpolymerized fraction. Pellets were sonicated with 8 M urea solution (8 M Urea/3 M Thiourea/4 % CHAPS/40 mM DTT) and used for polymerized fraction.

Lentiviral production and gene knockdown. Lentiviruses were produced in HEK293T cells via transfection with the following plasmids: pMD2.G (Addgene #12259, gift from Didier Trono), psPAX2 (Addgene #12260, gift from Didier Trono), and pLKO.1 shRNA expression vector (Addgene #8453, gift from Bob Weinberg).[4] shRNA vectors targeting *KRT18* and *shNT* non-targeting control vectors were constructed using the sequences shown in **Table S4**. A549 cells were infected with viruses and subjected to puromycin selection to isolate knockdown cells. Because KRT18 expression recovered soon after transduction, knockdown cells had to be used for assays within one week after selection.

Construction of lentiviral gene expression vectors. To express GFP-KRT18, LAMP1-GFP, and mCherry-LC3, pLentiN vector (Addgene #37444, gift from Karl Munger)[5] was used as a backbone vector for *GFP-KRT18* and *LAMP1-GFP* vectors. For *mCherry-LC3* vector,

the original blasticidin resistance marker gene was replaced with a puromycin resistance gene. Lentiviruses were produced as described above, using the pLKO.1 vector. Transfected cells were selected with corresponding antibiotics and single colonies were isolated.

Autophagic flux assay. To monitor autophagic flux and cell density, the pMRX-*IP-GFP-LC3-RFP-LC3ΔG* expression vector, a kind gift from Prof. Noboru Mizushima, was transfected stably into A549 cells.[6] The cells were treated with various concentrations of AZM, HCQ, or $BafA_1$, and the fluorescence intensity or cell density were monitored with IncuCyte ZOOM live-cell imaging system (Essen Bioscience). GFP/RFP fluorescence intensity was used to monitor autophagic flux.

Transmission electron microscopy (TEM). A549 cells were treated with 50 μM AZM for 24 h before being prepared for TEM observation as previously described.[7]

Tumor xenograft model. Animal protocols were in accordance with the Regulations and Guidelines on Scientific and Ethical Care and Use of Laboratory Animals by Science Council of Japan and were approved by the Tokyo Medical University Animal Care and

Use Committee. A549 cells were injected into six- to eight-week-old male nude mice (BALB/cAJcl-nu/nu). A total of 1 x 10^6 cells were suspended in PBS, mixed with an equal volume of Matrigel (CORNING, #354234), and injected subcutaneously into the flank. Tumor size was measured with calipers twice per week, and body weight of mice was measured every day. After the average tumor volume reached 100 mm^3, mice were divided into two groups so that the average size of the bearing tumors was the same (not randomized), and treated with either azithromycin (100 µg/g/day) or vehicle only (0.25 % (w/v) methyl cellulose/0.1% Tween 80) via oral gavage. After three weeks of treatment, tumors were excised and either stored at -80°C for protein extraction or fixed in 10% formalin neutral buffer solution. No power calculation for sample sizes was performed. No blinding treatment was done.

Immunohistochemical analysis. Immunohistochemical analysis of xenograft tumors was performed with anti-p62 (PROGEN, #GP62-C) antibody. Paraffin sections were deparaffinized and rehydrated. Antigen retrieval was performed by autoclaving at 121°C for 10 min in a retrieval solution (10 mM sodium citrate, pH 6.0). Subsequently, sections were blocked with 10% normal goat serum in TBST and incubated with primary antibody (1:1000) at 4°C for 16 h. For secondary antibody, anti-guinea pig Alexa488 conjugated

(Thermo #A11073, 1:200) was used, and DAPI was used for staining nuclei. To suppress autofluorescence, Vector TrueVIEW Autofluorescence Quenching Kit was used (VECTOR LABORATORIES #SP-8400). Stained sections were observed via confocal microscopy.

Confocal microscopic observation of live cells. Observation of A549 cells expressing either GFP-KRT18, LAMP1-EGFP, mCherry-LC3, or mCherry-EGFP-LC3 were performed using a confocal laser scanning fluorescence microscope, LSM 700 (Carl Zeiss). Cells were incubated at 37°C, under 5% CO_2 and humidified conditions. To analyze lysosomal proteolysis, A549 cells expressing LAMP1-EGFP were cultured in the presence of inhibitors for 24 h before being cultured in fresh medium containing 10 μg/mL DQ Red BSA (Thermo #D12051) and inhibitors for 6 h. Cells were subsequently washed twice with medium and replaced with either new medium or HBSS containing inhibitors. To measure the intracellular movement of lysosomes/autolysosomes, LAMP1-EGFP-expressing A549 cells were treated with 50 μM AZM or DMSO for 4 h, and time lapse images of LAMP1-EGFP positive vesicles were obtained every 2 s for a total of 200 s. For track analysis of LAMP1-EGFP positive vesicles, mean displacement and mean velocity of tracks were analyzed with TrackMate through Fiji from 10 different fields.[8, 9]

Statistical analysis. Statistical analysis for the FACS analysis and image analysis results in Fig. 3B performed a one-way ANOVA followed by Bonferroni's multiple comparison test. Two-sided Student’s t-test was performed to compare tumor weight, tracking analysis results, DQ Red-BSA analysis, and image analysis results in Fig. 3D. A *p*-value of less than 0.05 was considered statistically significant. Variation across experimental groups was analyzed using F-testing. All analyses were performed with GraphPad Prism 5 software (GraphPad Software).

References

1 Saito Y, Moriya S, Kazama H, Hirasawa K, Miyahara K, Kokuba H, et al. Amino acid starvation culture condition sensitizes EGFR-expressing cancer cell lines to gefitinib-mediated cytotoxicity by inducing atypical necroptosis. *International journal of oncology*. 2018; 52: 1165-1177.

2 Yamada S, Wirtz D, Coulombe PA. Pairwise assembly determines the intrinsic potential for self-organization and mechanical properties of keratin filaments. *Mol Biol Cell*. 2002; 13: 382-391.

3 Hood KA, West LM, Rouwé B, Northcote PT, Berridge MV, Wakefield SJ, et al. Peloruside A, a novel antimitotic agent with paclitaxel-like microtubule- stabilizing activity. *Cancer Res*. 2002; 62: 3356-3360.

4 Stewart SA, Dykxhoorn DM, Palliser D, Mizuno H, Yu EY, An DS, et al. Lentivirus-delivered stable gene silencing by RNAi in primary cells. *RNA*. 2003; 9: 493-501.

5 Spangle JM, Ghosh-Choudhury N, Munger K. Activation of cap-dependent translation by mucosal human papillomavirus E6 proteins is dependent on the integrity of the LXXLL binding motif. *J Virol*. 2012; 86: 7466-7472.

6 Kaizuka T, Morishita H, Hama Y, Tsukamoto S, Matsui T, Toyota Y, et al. An autophagic flux probe that releases an internal control. *Mol Cell*. 2016; 64: 835-849.

7 Takeda A, Takano N, Kokuba H, Hino H, Moriya S, Abe A, et al. Macrolide antibiotics enhance the antitumor effect of lansoprazole resulting in lysosomal membrane permeabilization-associated cell death. *International journal of oncology*. 2020; 57: 1280-1292.

8 Schindelin J, Arganda-Carreras I, Frise E, Kaynig V, Longair M, Pietzsch T, et al. Fiji: an open-source platform for biological-image analysis. *Nat Methods*. 2012; 9: 676-682.

9 Tinevez JY, Perry N, Schindelin J, Hoopes GM, Reynolds GD, Laplantine E, et al. TrackMate: An open and extensible platform for single-particle tracking. *Methods*. 2017; 115: 80-90.
